# Supplementary material for: Design, Synthesis, Antibacterial, and Antifungal Evaluation of Phenylthiazole Derivatives Containing a 1,3,4-Thiadiazole Thione Moiety
Source: Molecules. 2024 Jan 5;29(2):285. doi: 10.3390/molecules29020285 (PMC10820687; doi:10.3390/molecules29020285)
Supplement: Supplementary file 1 [file molecules-29-00285-s001.zip › molecules-2807198-supplementary.pdf]

Supporting Information

Design, Synthesis, Antibacterial, and Antifungal Evaluation of Phenylthiazole Derivatives Containing a 1,3,4-Thiadiazole Thione Moiety

Guoqing Mao[1,2†], Yao Tian [1,2†], Jinchao Shi [1,2], Changzhou Liao[1,2], Weiwei Huang[1,2], Yiran Wu[1,2], Zhou Wen[1,2], Linhua Yu [1,2], Xiang Zhu [1,2,3,*] and Junkai Li [1,2,*]

1 Engineering Research Center of Ecology and Agricultural Use of Wetland, Ministry of Education, Hubei Key Laboratory of Waterlogging Disaster and Agricultural Use of Wetland, College of Agriculture, Yangtze University, Jingzhou 434025, China; maoguoqing1688@163.com (G.M.); yaotien@163.com (Y.T.); shijinchao1996@163.com (J.S.); liaochangzhou58@163.com (C.L.); 18381914696@163.com (W.H.); wu18230085332@163.com (Y.W.); wenzhou1218@163.com (Z.W.); linhuayu531@sina.com (L.Y.)

2 Institute of Pesticides, Yangtze University, Jingmi Road 88, Jingzhou 434025, China

3 National Key Laboratory of Green Pesticide, Key Laboratory of Green Pesticide and Agricultural Bioengineering, Ministry of Education, Guizhou University, Guiyang 550025, China

* Correspondence: xiangzhu1992@yangtzeu.edu.cn (X.Z.); junkaili@sina.com (J.L.)

† These two authors contributed equally to this work.

Table of Contents

1. The structure and data of target compounds (**5a**-**5p**) **2**

Compound **5a**, 5-(2-phenylthiazol-4-yl)-1,3,4-thiadiazole-2(3*H*)-thione:

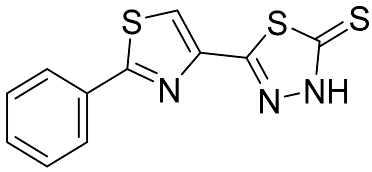

white solid, yield 83.2 %, m. p. >250°C; ^{1}H NMR (400 MHz, DMSO-d_6) δ: 14.45 (s, 1H), 8.43 (s, 1H), 8.02 – 7.87 (m, 2H), 7.62 – 7.44 (m, 3H). ^{13}C NMR (101 MHz, DMSO-d_6) δ: 188.38, 169.10, 155.37, 144.85, 132.30, 131.53, 129.85 (2C), 126.86 (2C), 120.54. HRMS (ESI): calcd for $C_{11}H_7N_3S_3$ $[M+H]^+$: 277.9875, found, 277.9875.

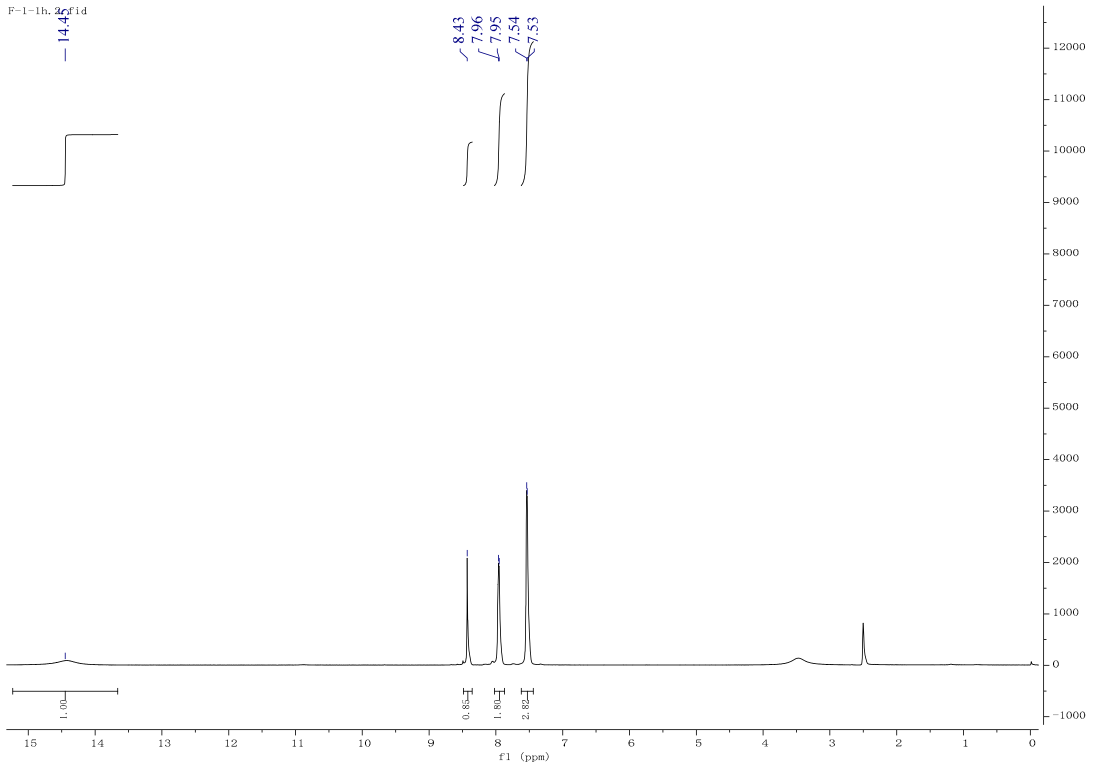

The ^{1}H NMR spectrogram of compound **5a**

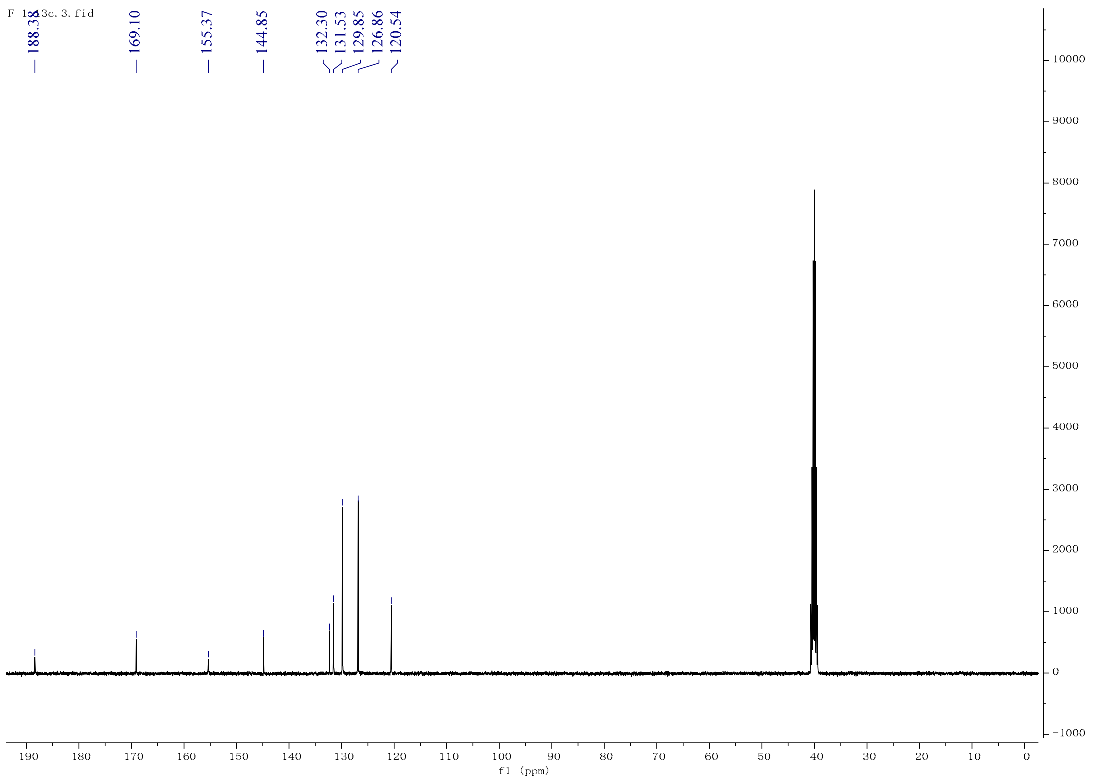

The ^{13}C NMR spectrogram of compound **5a**

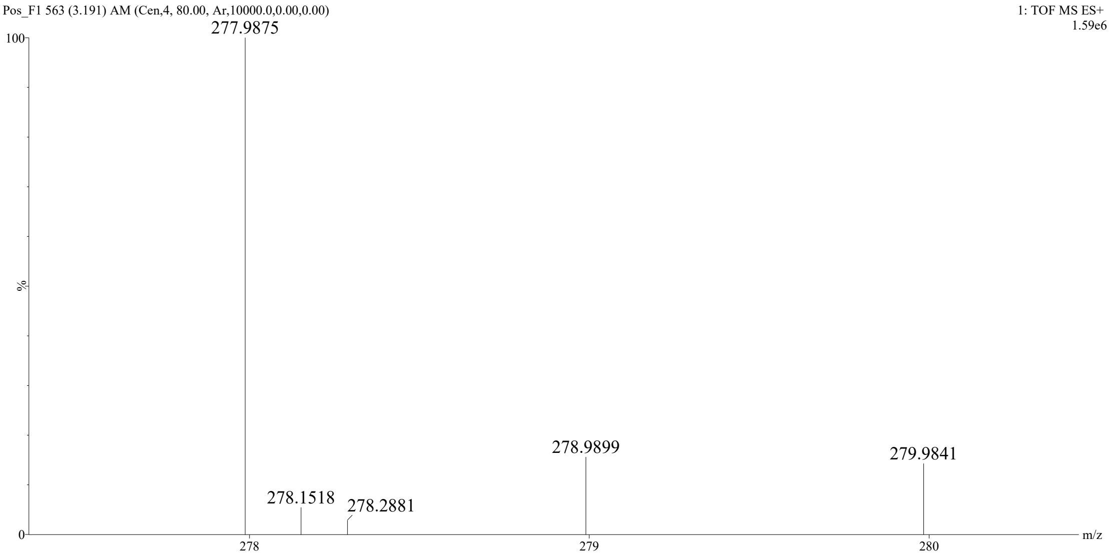

The HRMS spectrogram of compound **5a**

Compound **5b**, 5-(2-(2-fluorophenyl)thiazol-4-yl)-1,3,4-thiadiazole-2(3*H*)-thione:

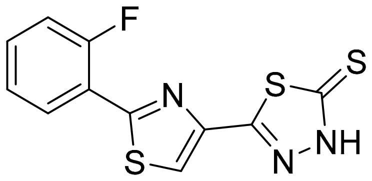

white solid, yield 84.7 %, m. p. 238.5-239.5°C; ^{1}H NMR (400 MHz, DMSO-d_6) δ: 17.37 (s, 1H), 11.20 (s, 1H), 10.77 (t, J = 7.8 Hz, 1H), 10.12 (q, J = 7.0 Hz, 1H), 10.03 – 9.86 (m, 2H). ^{13}C NMR (101 MHz, DMSO-d_6) δ: 177.23, 161.46 (d, J = 5.0 Hz), 159.46 (d, J = 250.8 Hz), 156.44, 138.05, 132.81 (d, J = 8.8 Hz), 128.46, 125.51 (d, J = 9.1 Hz), 125.41 (d, J = 3.2 Hz), 119.59 (d, J = 11.4 Hz), 116.50 (d, J = 21.3 Hz). HRMS (ESI): calcd for $C_{11}H_6FN_3S_3$ [M+H]$^+$: 295.9781, found, 295.9781.

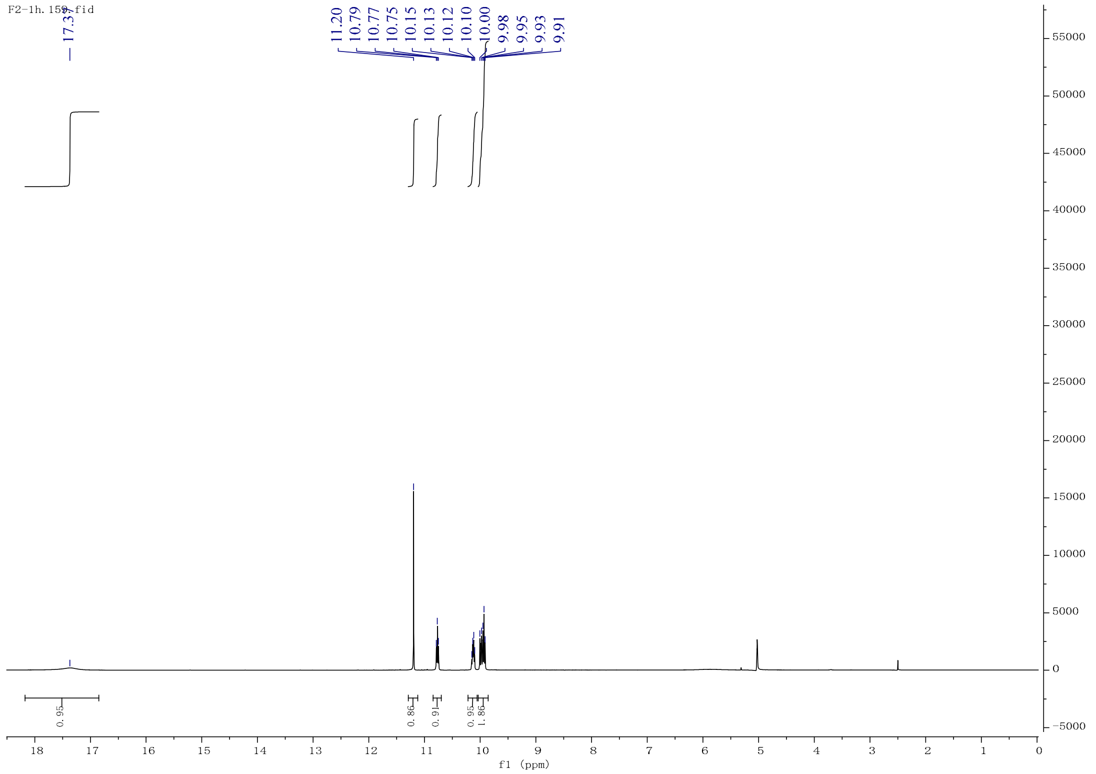

The ^{1}H NMR spectrogram of compound **5b**

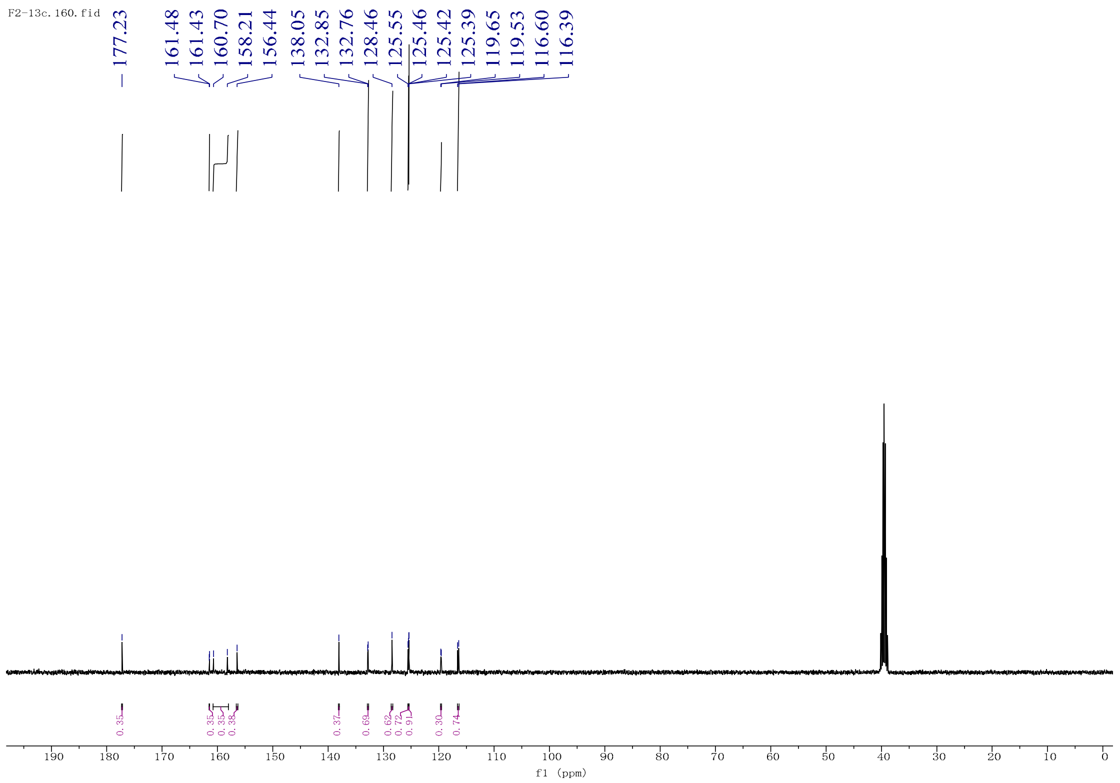

The ^{13}C NMR spectrogram of compound **5b**

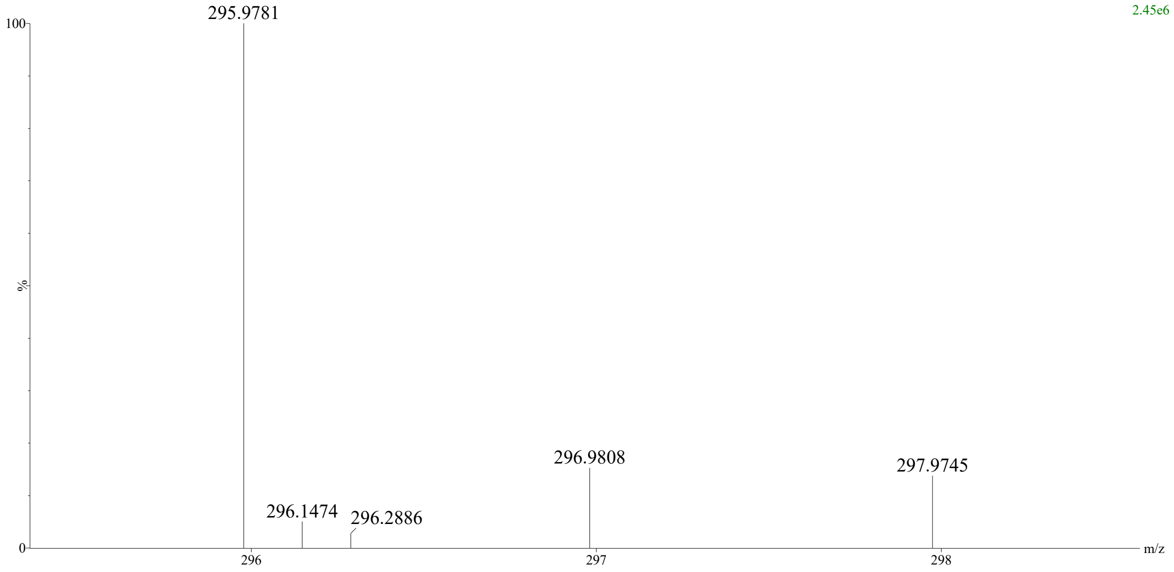

The HRMS spectrogram of compound **5b**

Compound **5c**, 5-(2-(2-chlorophenyl)thiazol-4-yl)-1,3,4-thiadiazole-2(3*H*)-thione:

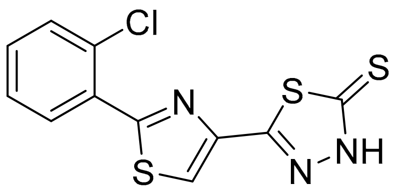

white solid, yield 80.1 %, m. p. 234.6-234.9°C; ^{1}H NMR (400 MHz, DMSO-d_6) δ: 14.70 (s, 1H), 8.58 (s, 1H), 8.18 (dd, J = 6.0, 3.6 Hz, 1H), 7.74 – 7.63 (m, 1H), 7.55 (p, J = 6.4 Hz, 2H). ^{13}C NMR (101 MHz, DMSO-d_6) δ: 189.48, 164.30, 160.39, 143.87, 132.38, 131.40, 131.32, 131.19, 130.51, 128.44, 122.03. HRMS (ESI): calcd for $C_{11}H_6ClN_3S_3$ [M+H]$^+$: 311.9485; found, 311.9487.

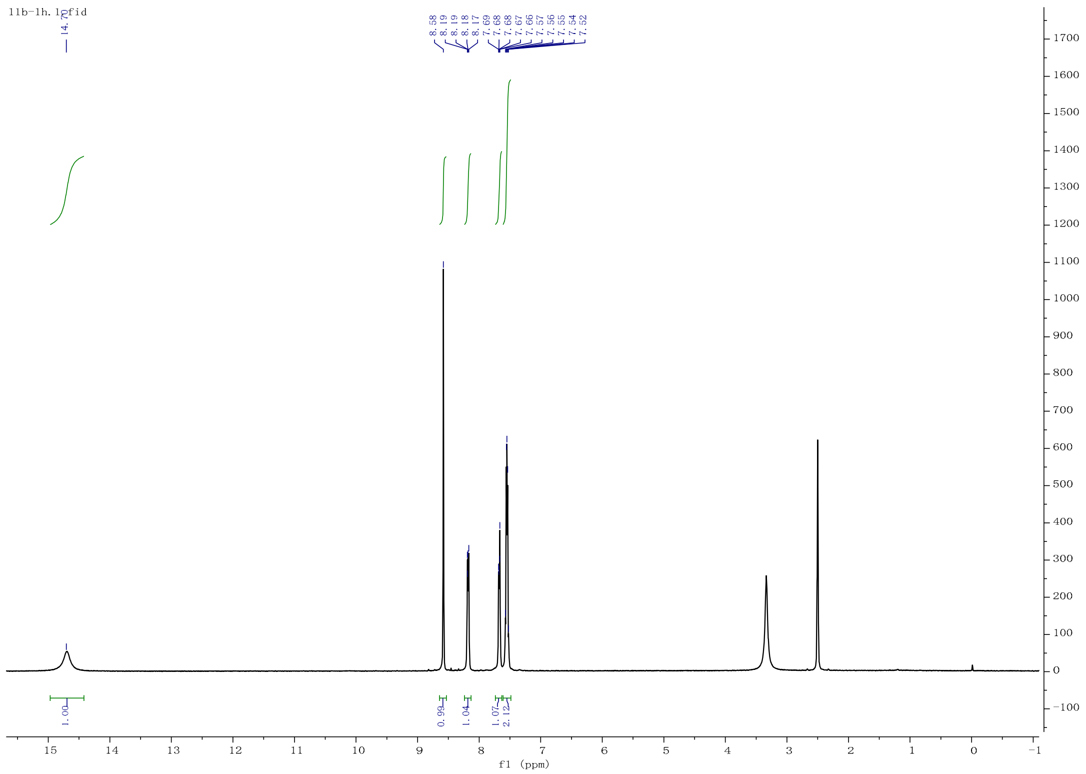

The ^{1}H NMR spectrogram of compound **5c**

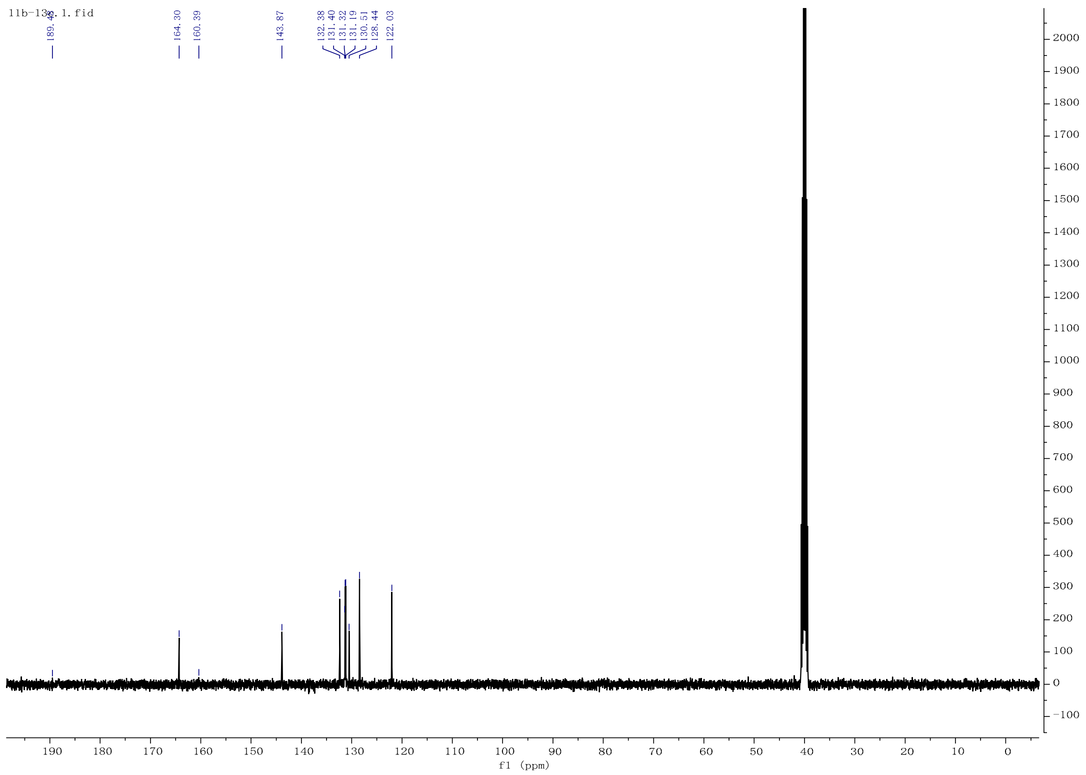

The ^{13}C NMR spectrogram of compound **5c**

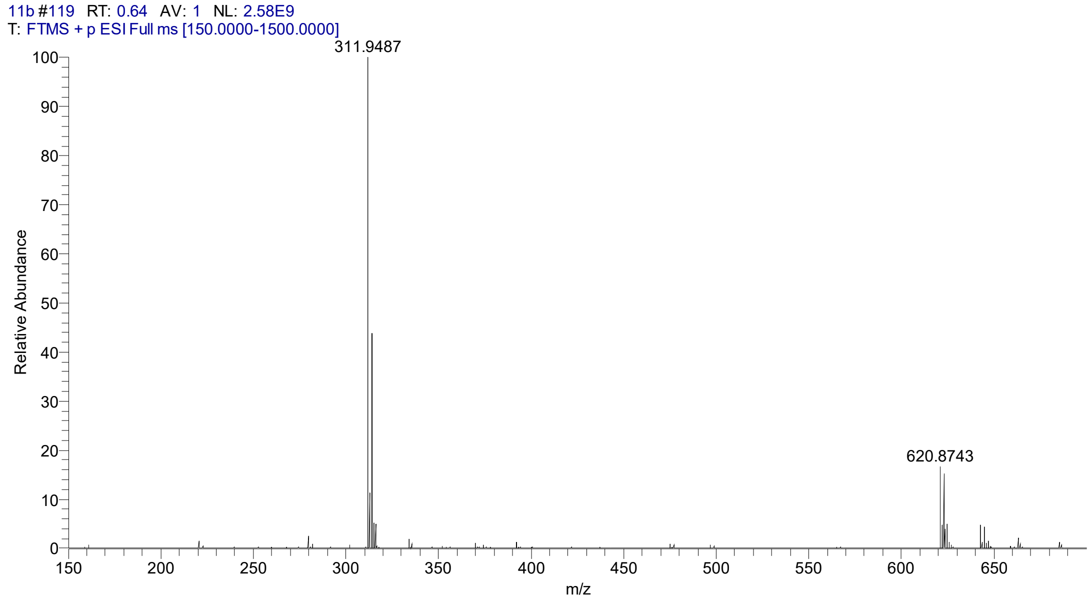

The HRMS spectrogram of compound **5c**

Compound **5d**, 5-(2-(2-bromophenyl)thiazol-4-yl)-1,3,4-thiadiazole-2(3*H*)-thione:

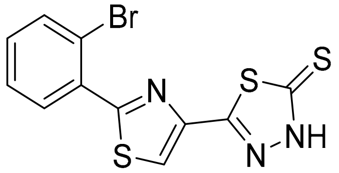

yellow solid, yield 84.3%, m. p. 222.7-223.7°C; ^{1}H NMR (400 MHz, DMSO-d_6) δ: 14.24 (s, 1H), 8.56 (d, J = 2.5 Hz, 1H), 8.03 (d, J = 7.8 Hz, 1H), 7.82 (d, J = 7.9 Hz, 1H), 7.55 (t, J = 7.5 Hz, 1H), 7.45 (t, J = 7.6 Hz, 1H). ^{13}C NMR (101 MHz, DMSO-d_6) δ: 188.00, 165.95, 155.55, 143.98, 134.61, 132.66, 132.44, 131.95, 128.77, 121.92, 121.35. HRMS (ESI): calcd for $C_{11}H_6BrN_3S_3$ $[M+H]^+$: 355.8980, found, 355.8981.

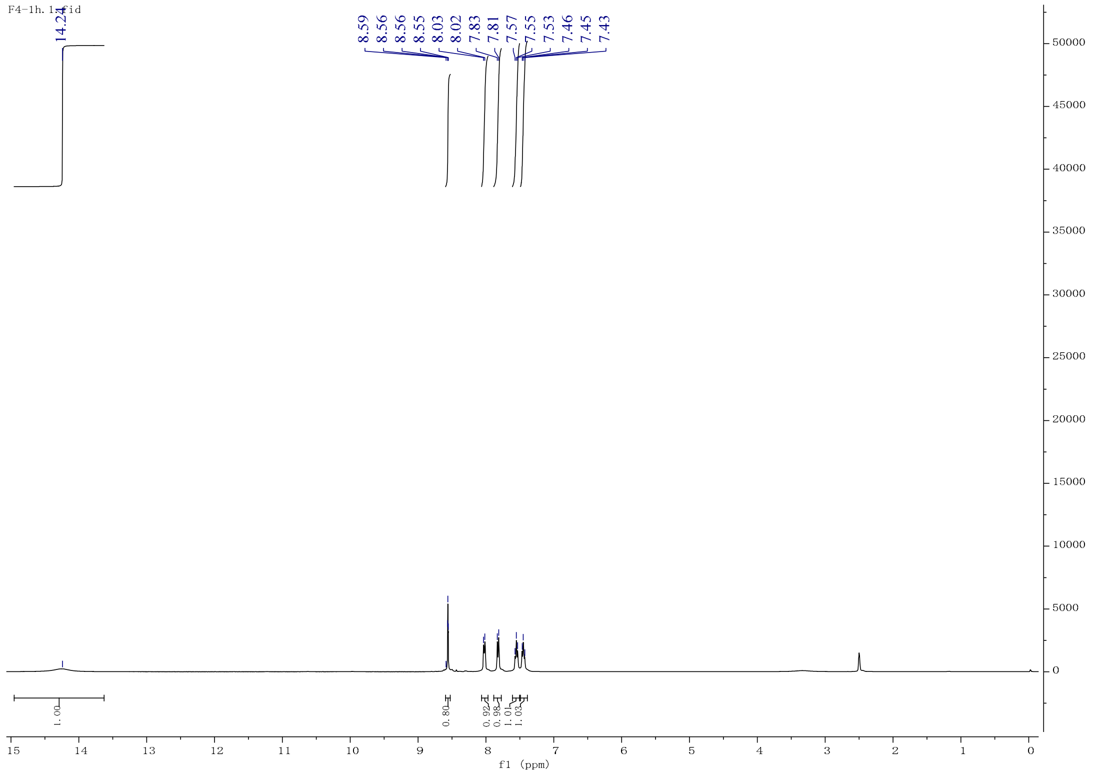

The ^{1}H NMR spectrogram of compound **5d**

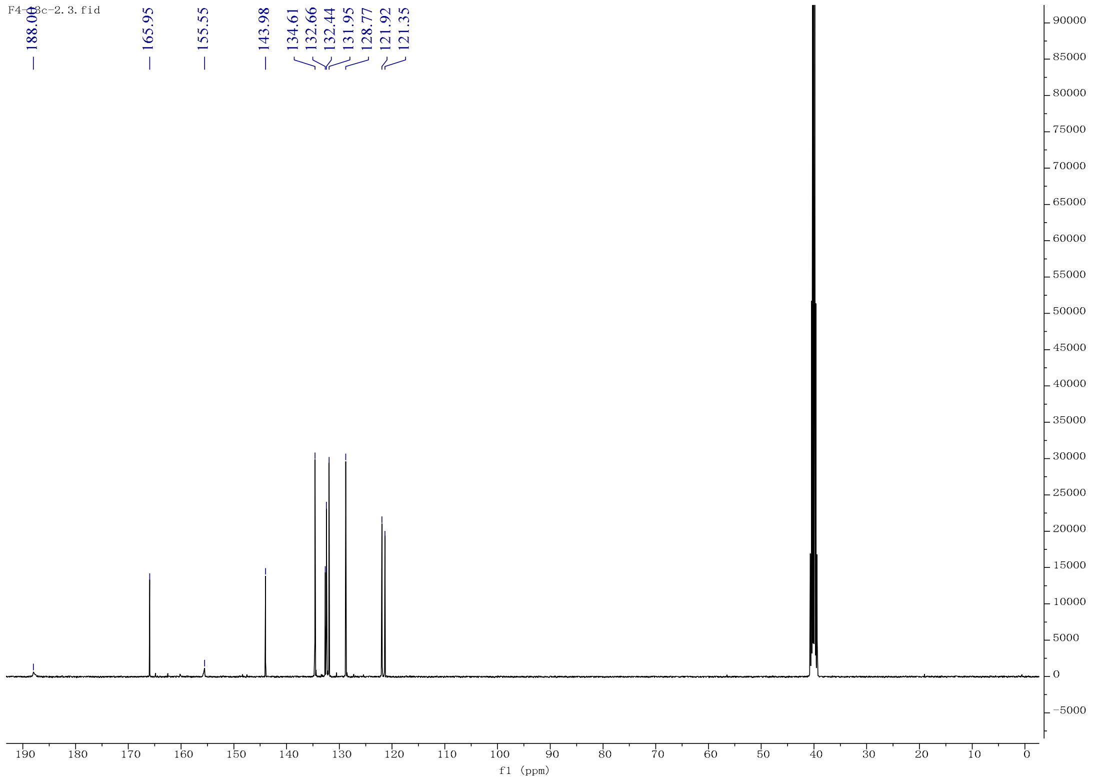

The ^{13}C NMR spectrogram of compound **5d**

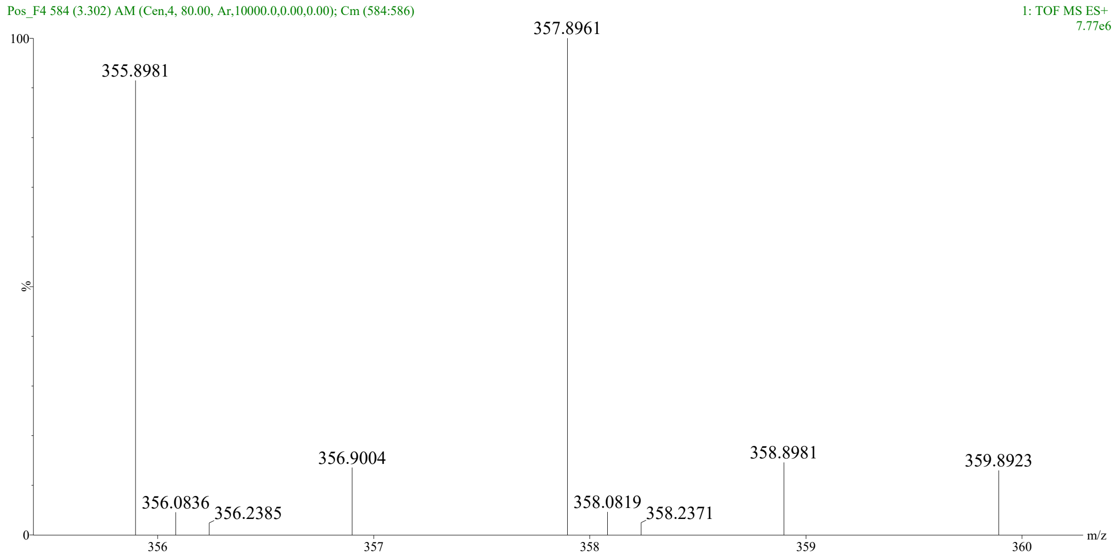

The HRMS spectrogram of compound **5d**

Compound **5e**, 5-(2-(o-tolyl)thiazol-4-yl)-1,3,4-thiadiazole-2(3*H*)-thione:

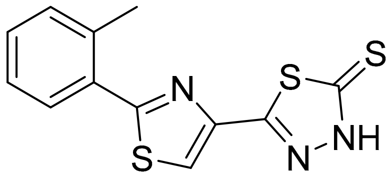

white solid, yield 83.2%, m. p. 229.3-230.1°C; ^{1}H NMR (400 MHz, DMSO-*d*$_6$) δ: 14.78 (s, 1H), 8.51 (s, 1H), 7.78 (d, *J* = 7.2 Hz, 1H), 7.52 – 7.29 (m, 3H), 3.32 (s, 3H). ^{13}C NMR (101 MHz, DMSO-*d*$_6$) δ: ^{13}C NMR (101 MHz, DMSO-*d*$_6$) δ: 188.36, 168.85, 155.74, 144.27, 136.62, 132.26, 131.49, 130.77, 130.10, 127.05, 120.66, 21.70. HRMS (ESI): calcd for $C_{12}H_9N_3S_3$ [M+H]$^+$: 292.0031, found, 292.0032.

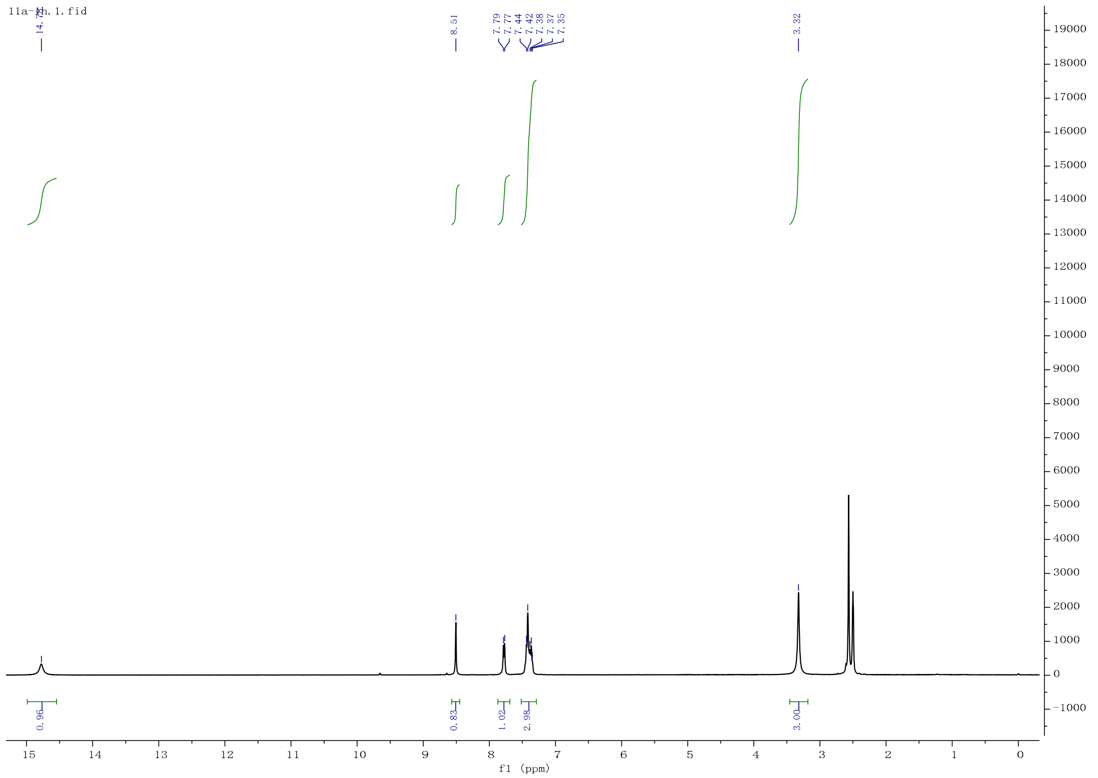

The ^{1}H NMR spectrogram of compound **5e**

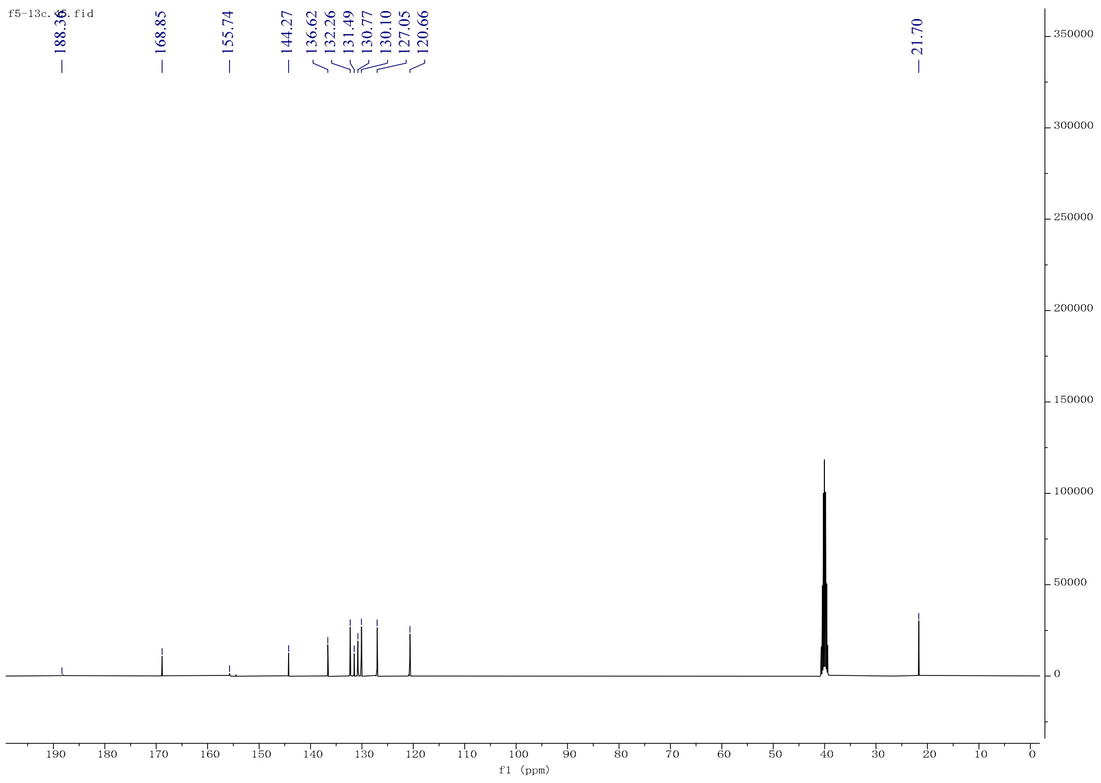

The ^{13}C NMR spectrogram of compound **5e**

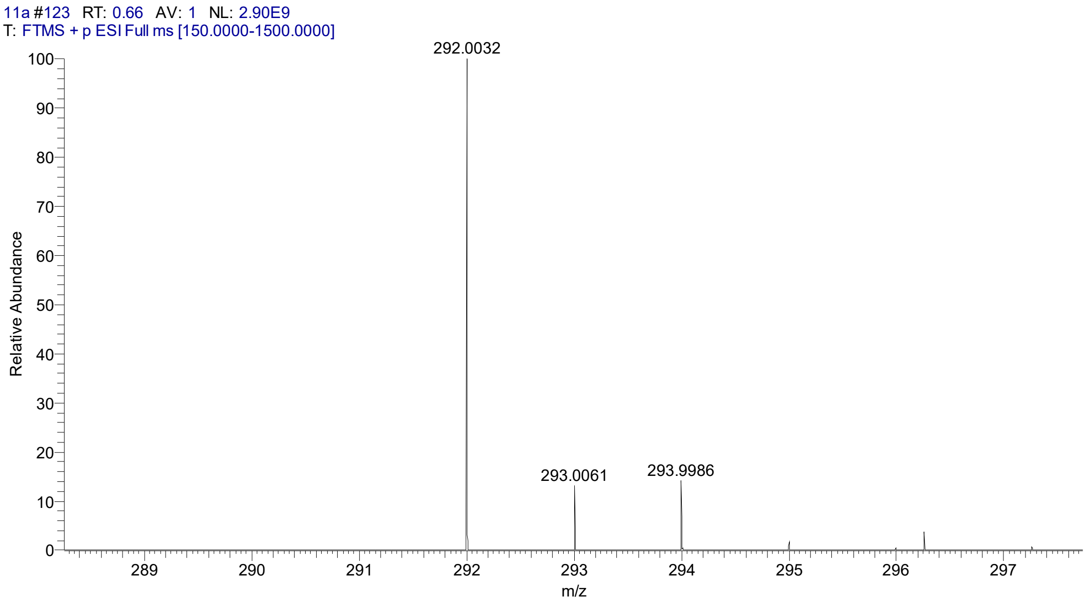

The HRMS spectrogram of compound **5e**

Compound **5f**, 5-(2-(2-methoxyphenyl)thiazol-4-yl)-1,3,4-thiadiazole-2(3*H*)-thione:

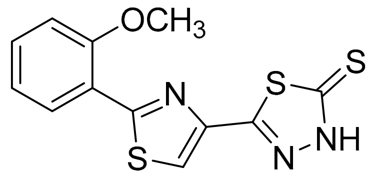

white solid, yield 82.7%, m. p.235.6-236.7C; ^{1}H NMR (400 MHz, DMSO-d_6) δ: 13.71 (s, 1H), 8.39 (s, 1H), 8.23 (d, J = 7.8 Hz, 1H), 7.50 (t, J = 7.8 Hz, 1H), 7.25 (d, J = 8.4 Hz, 1H), 7.13 (t, J = 7.6 Hz, 1H), 4.02 (s, 3H). ^{13}C NMR (101 MHz, DMSO-d_6) δ: 188.07, 163.06, 156.73, 147.66, 143.19, 132.41, 127.97, 121.52, 120.84, 120.65, 112.78, 56.47. HRMS (ESI): calcd for $C_{12}H_9N_3OS_3$ [M+H]$^+$: 307.9981, found, 307.9983.

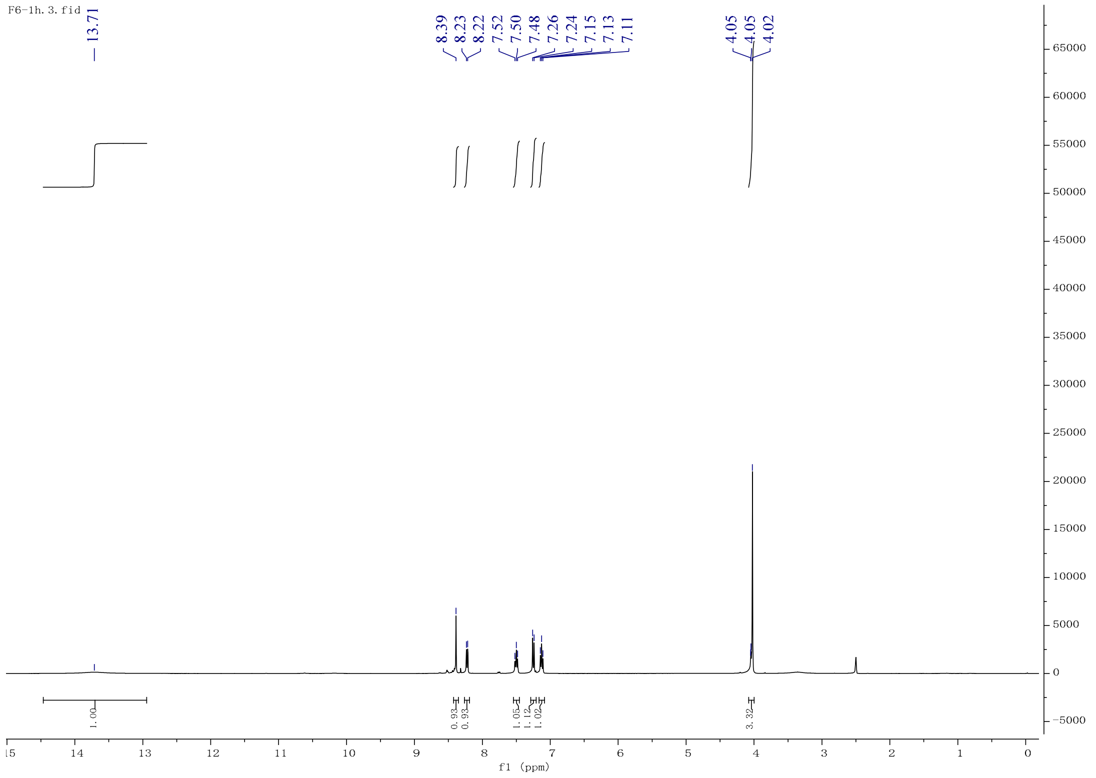

The ^{1}H NMR spectrogram of compound **5f**

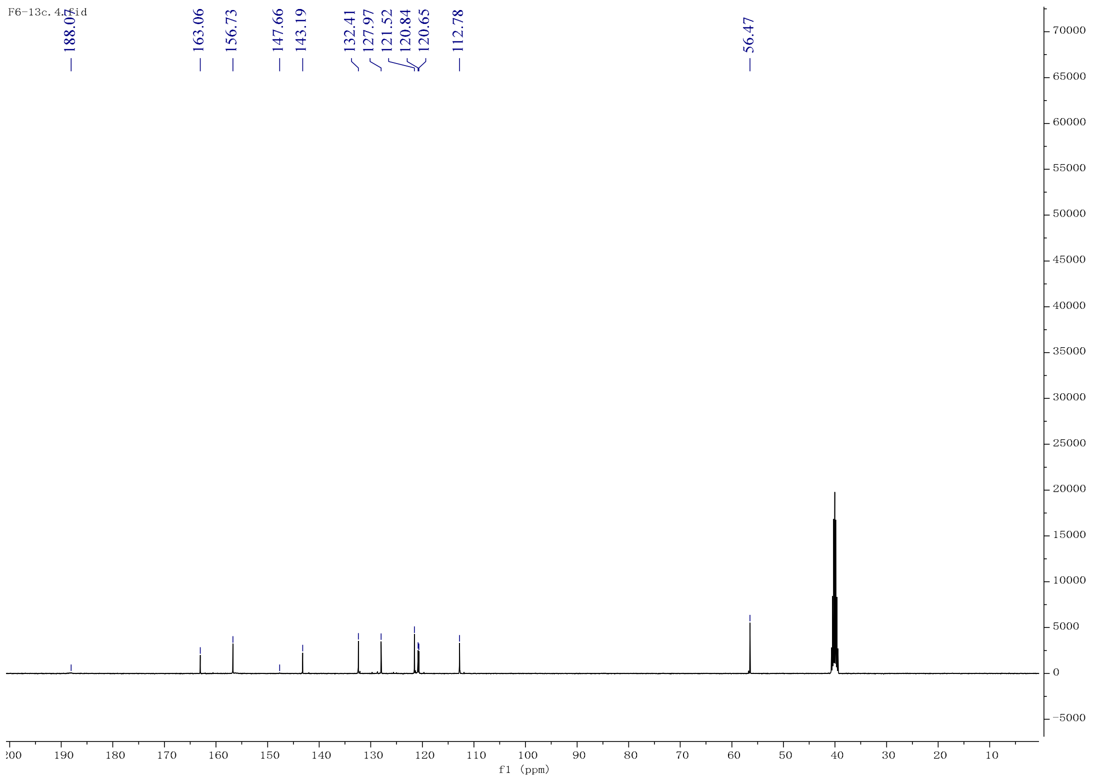

The ^{13}C NMR spectrogram of compound **5f**

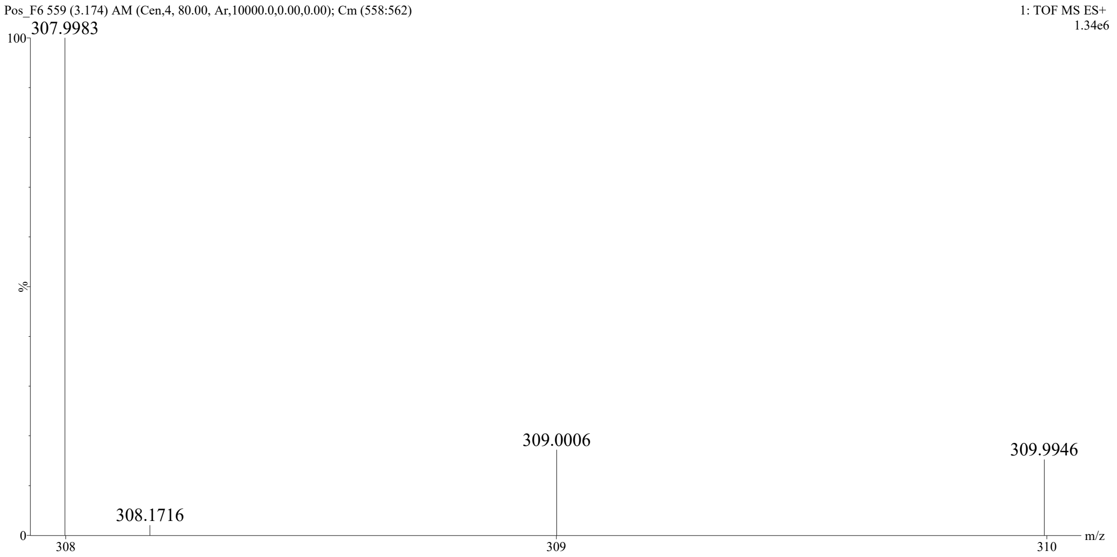

The HRMS spectrogram of compound **5f**

Compound **5g**, 5-(2-(2-(trifluoromethoxy)phenyl)thiazol-4-yl)-1,3,4-thiadiazole-2(3*H*)

-thione:

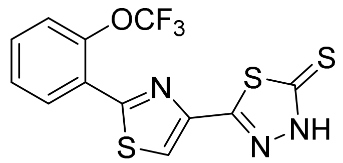

white solid, yield 85.6%, m. p. 230.2-230.7℃; ^{1}H NMR (400 MHz, DMSO-d_6) δ: 14.83 (s, 1H), 8.60 (s, 1H), 8.30 (dd, J = 8.1, 1.7 Hz, 1H), 7.73 – 7.67 (m, 1H), 7.65 – 7.59 (m, 2H). ^{13}C NMR (101 MHz, DMSO-d_6) δ: 187.88, 161.40, 154.78, 145.14, 143.65, 132.39, 129.62, 128.13, 124.58, 121.56, 121.12, 120.01 (q, J = 259.1 Hz). HRMS (ESI): calcd for $C_{12}H_6F_3N_3OS_3$ [M+H]$^+$: 361.9698, found, 361.9697.

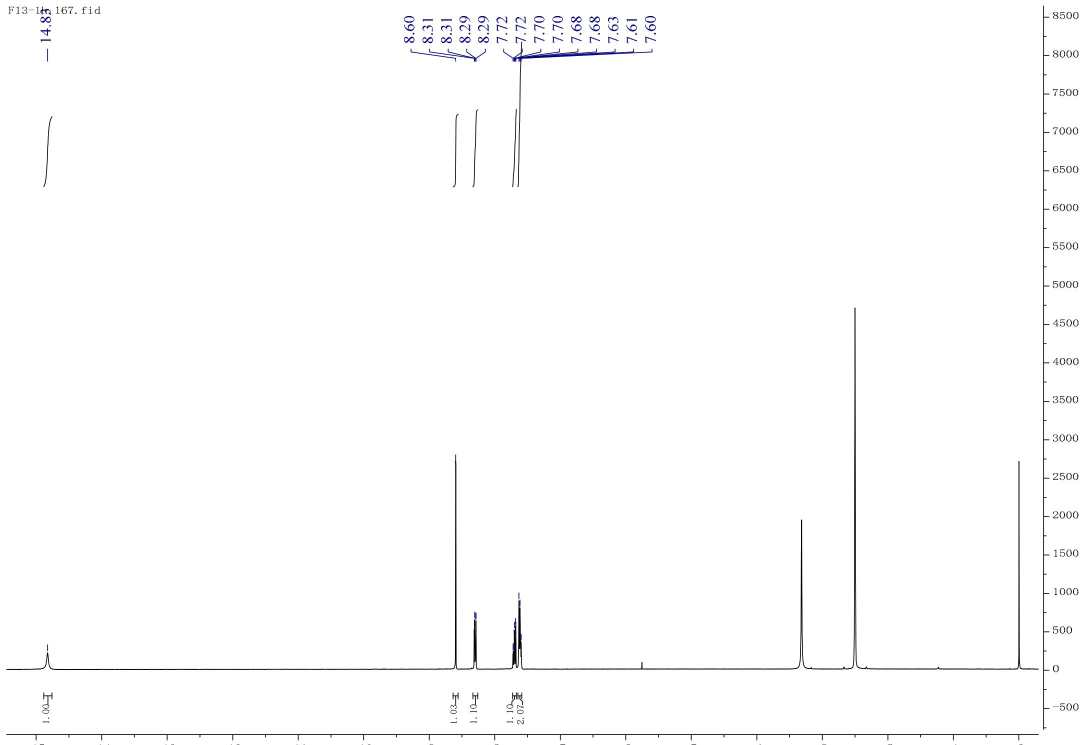

The ^{1}H NMR spectrogram of compound **5g**

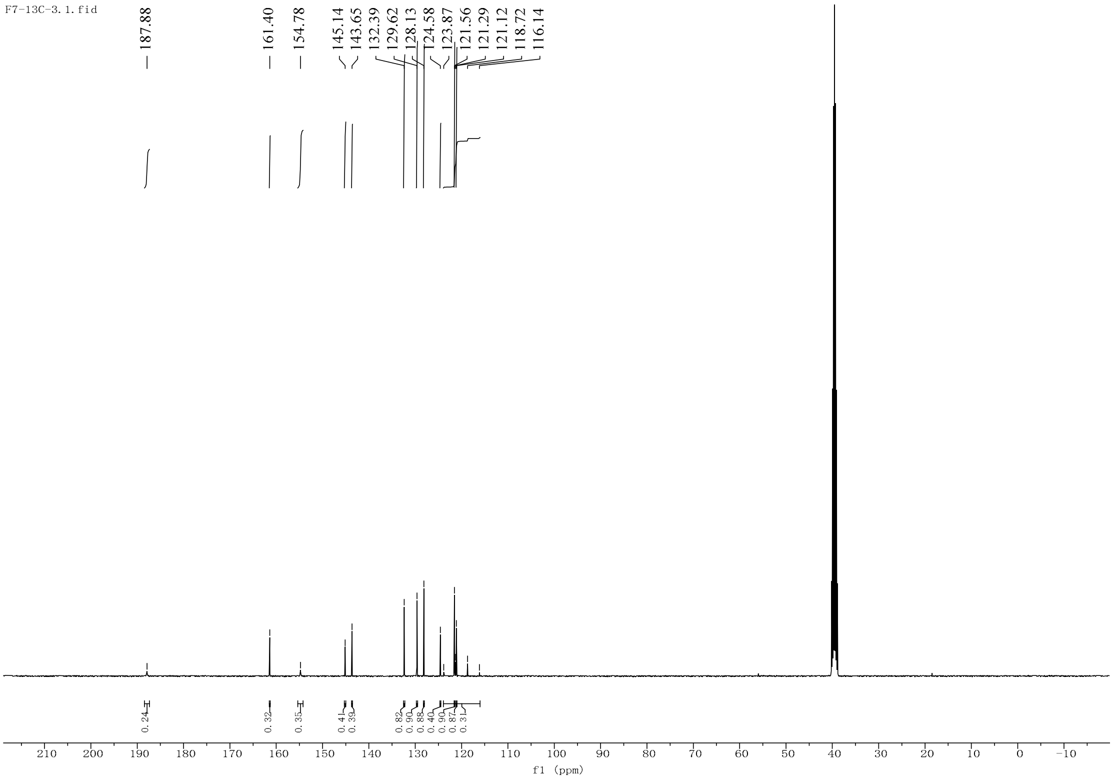

The ^{13}C NMR spectrogram of compound **5g**

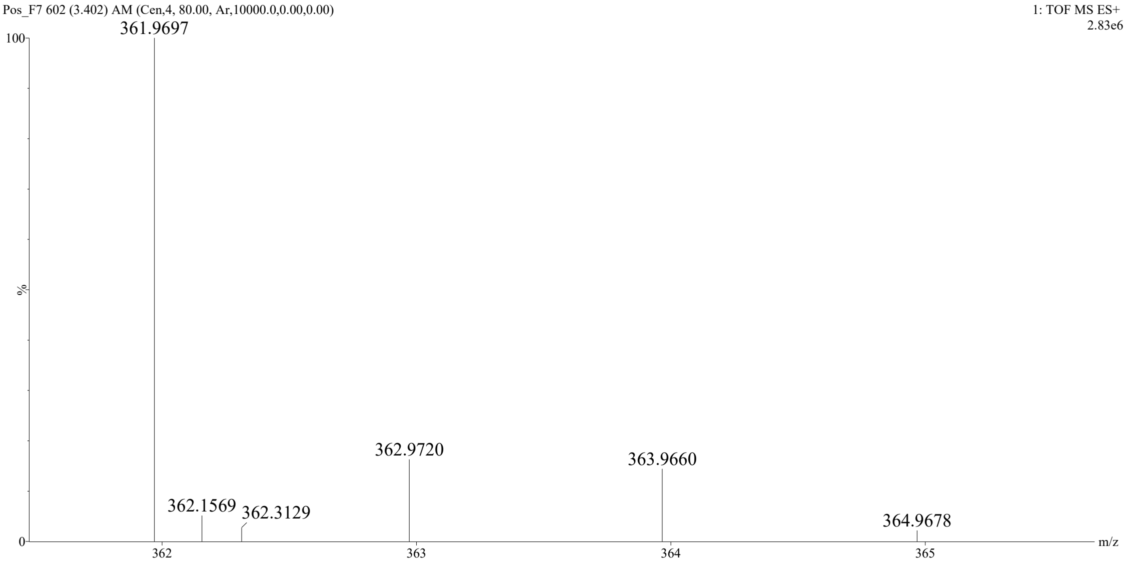

The HRMS spectrogram of compound **5g**

Compound **5h**, 5-(2-(3-fluorophenyl)thiazol-4-yl)-1,3,4-thiadiazole-2(3*H*)-thione:

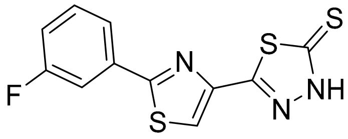

white solid, yield 84.5%, m. p. 219.1-220.0°C; ^{1}H NMR (400 MHz, DMSO-*d*$_6$) *δ*: 14.74 (s, 1H), 8.47 (s, 1H), 7.78 (dd, *J* = 17.5, 8.7 Hz, 2H), 7.68 – 7.52 (m, 1H), 7.39 (t, *J* = 8.3 Hz, 1H). ^{13}C NMR (101 MHz, DMSO-*d*$_6$) *δ*: 187.90, 167.02 (d, *J* = 3.1 Hz), 162.41 (d, *J* = 245.1 Hz), 154.72, 144.39, 133.88 (d, *J* = 8.0 Hz), 131.60 (d, *J* = 8.5 Hz), 122.66 (d, *J* = 2.6 Hz), 120.83, 117.82 (d, *J* = 21.1 Hz), 112.89 (d, *J* = 23.6 Hz). HRMS (ESI): calcd for $C_{11}H_6FN_3S_3$ [M+H]$^+$: 295.9781, found, 295.9782.

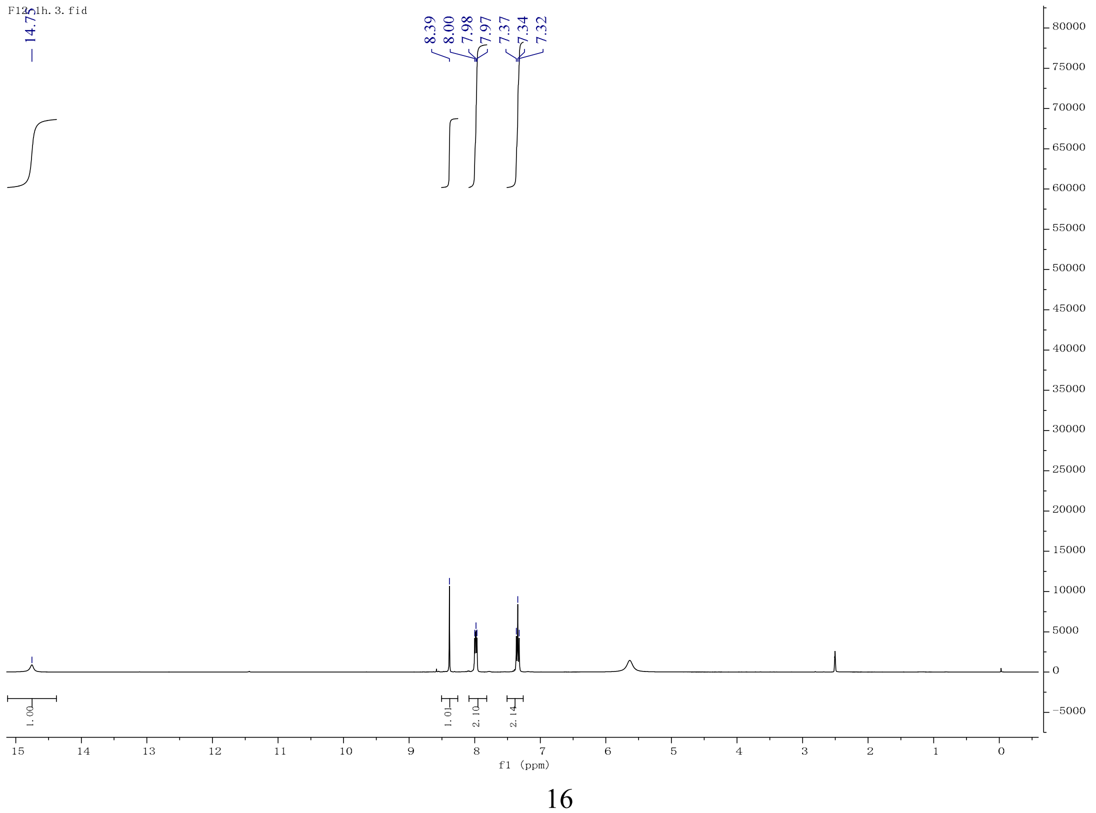

The ^{1}H NMR spectrogram of compound **5h**

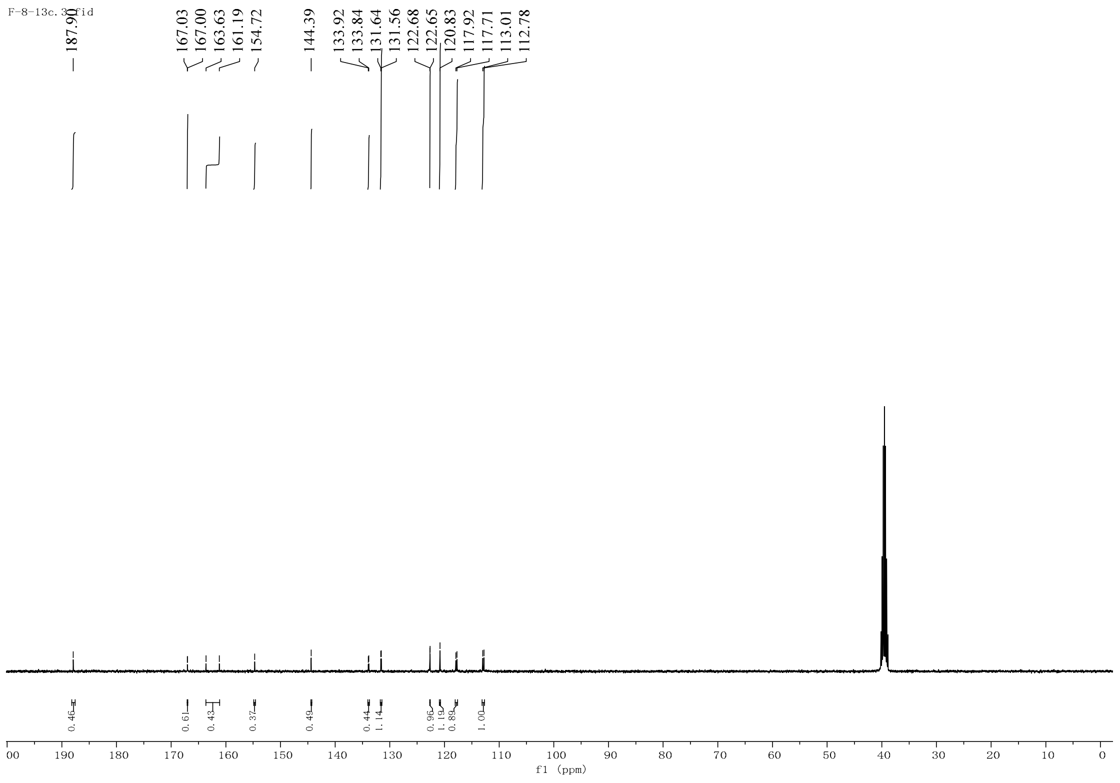

The ^{13}C NMR spectrogram of compound **5h**

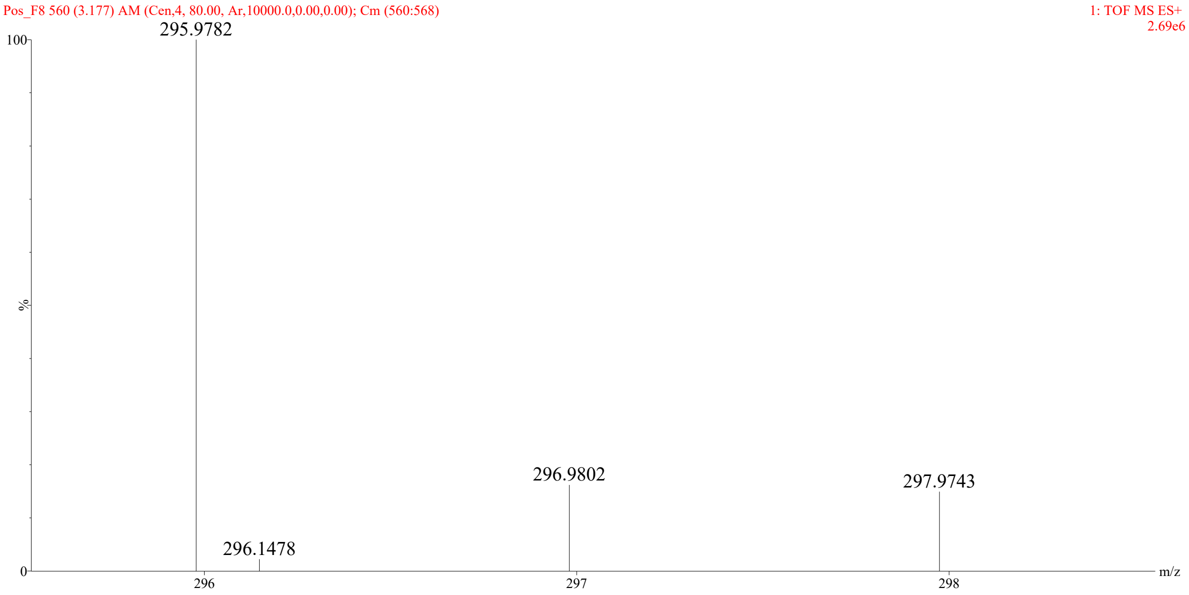

The HRMS spectrogram of compound **5h**

Compound **5i**, 5-(2-(m-tolyl)thiazol-4-yl)-1,3,4-thiadiazole-2(3*H*)-thione:

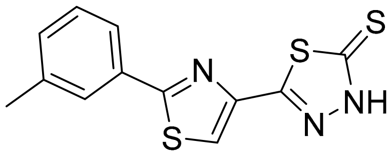

white solid, yield 84.8%, m. p. 224.9-225.4°C; ^{1}H NMR (400 MHz, DMSO-d_6) δ: 14.76 (s, 1H), 8.39 (s, 1H), 7.73 (d, J = 8.4 Hz, 2H), 7.45 – 7.28 (m, 2H), 2.38 (s, 3H). ^{13}C NMR (101 MHz, DMSO-d_6) δ: 188.33, 169.19, 155.41, 144.76, 139.28, 132.22, 132.20, 129.72, 127.16, 124.06, 120.39, 21.33. HRMS (ESI): calcd for $C_{12}H_9N_3S_3$

[M+H]$^+$: 292.0032, found, 292.0031.

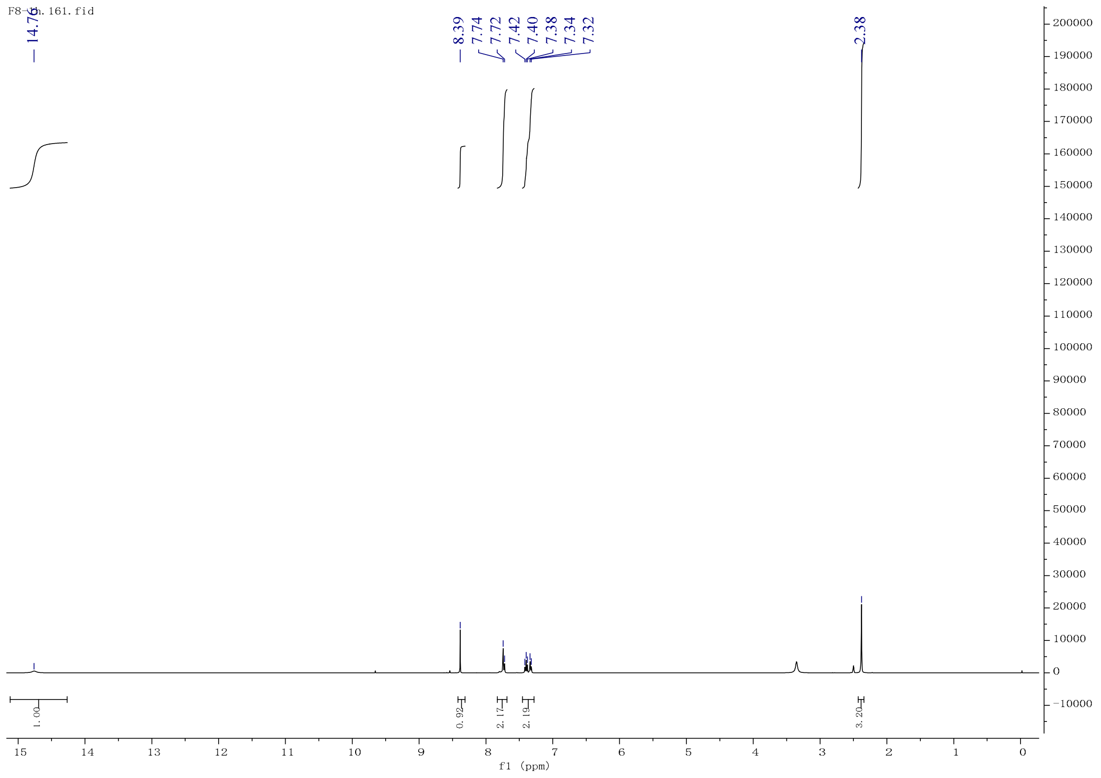

The ^{1}H NMR spectrogram of compound **5i**

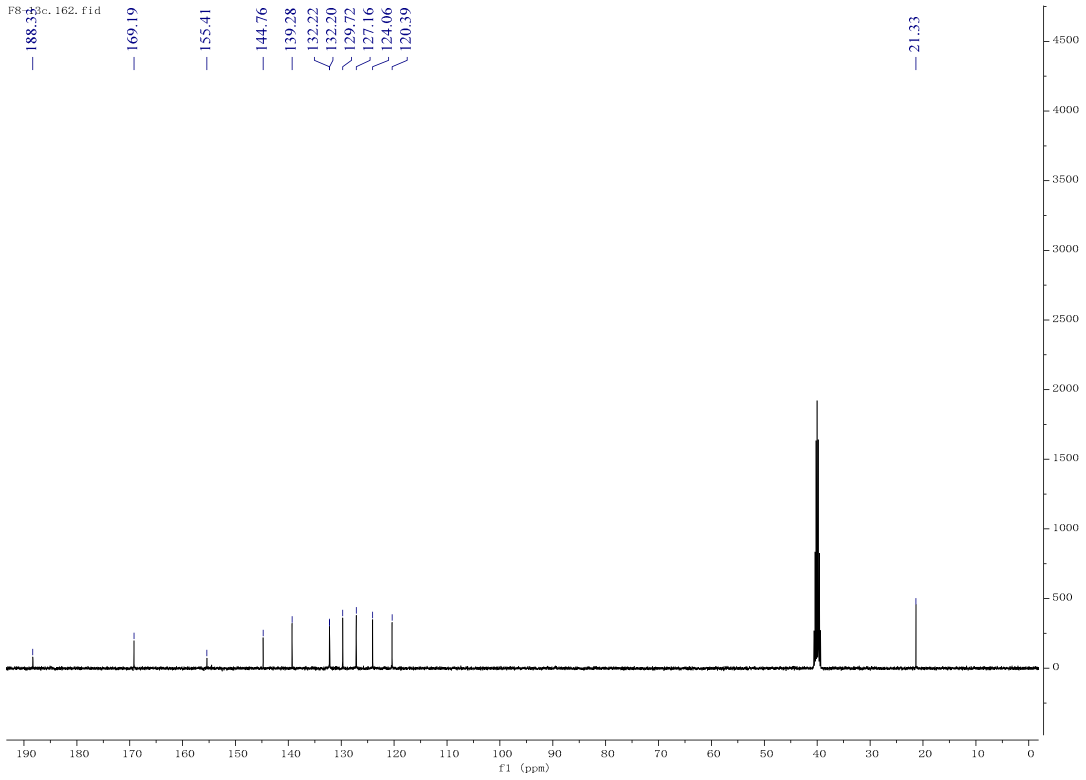

The ^{13}C NMR spectrogram of compound **5i**

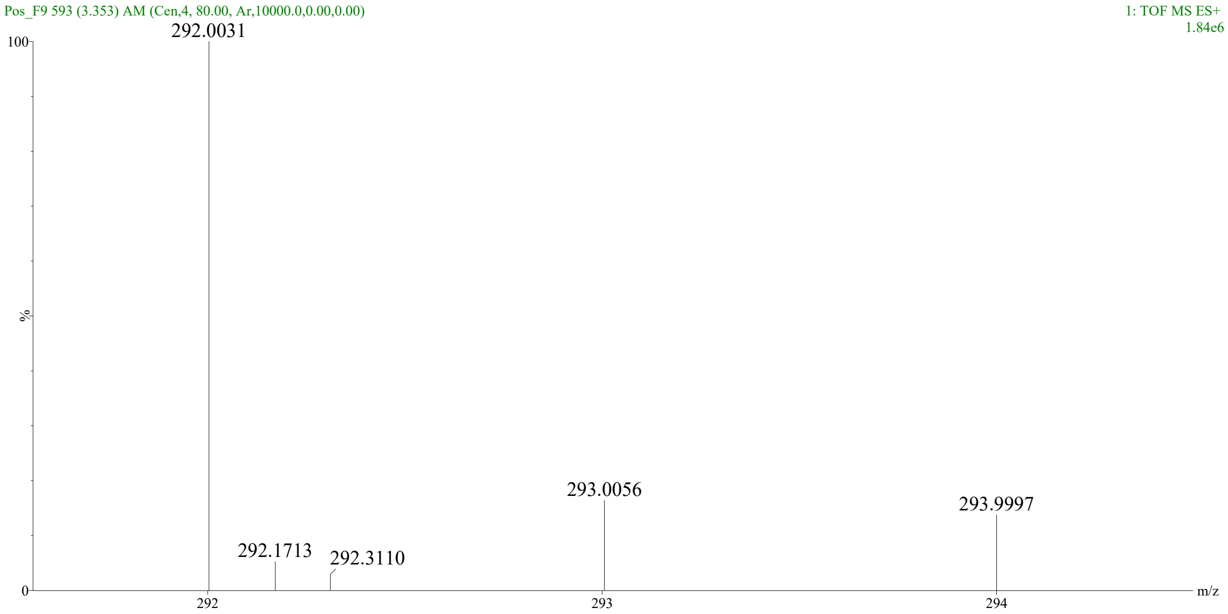

The HRMS spectrogram of compound **5i**

Compound **5j**, 5-(2-(3-methoxyphenyl)thiazol-4-yl)-1,3,4-thiadiazole-2(3*H*)-thione:

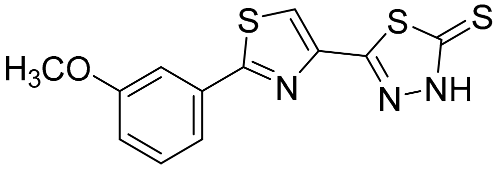

white solid, yield 83.1%, m. p. 228.9-230.1°C; ^{1}H NMR (400 MHz, DMSO-d_6) δ: 14.57 (s, 1H), 8.45 (s, 1H), 7.63 – 7.38 (m, 3H), 7.12 (d, *J* = 6.5 Hz, 1H), 3.85 (s, 3H). ^{13}C NMR (101 MHz, DMSO-d_6) δ: 188.40, 168.85, 160.23, 153.27, 144.78, 133.53, 131.11, 120.77, 119.32, 117.21, 111.83, 55.86. HRMS (ESI): calcd for $C_{12}H_9N_3OS_3$ [M+H]+: 307.9981, found, 307.9981.

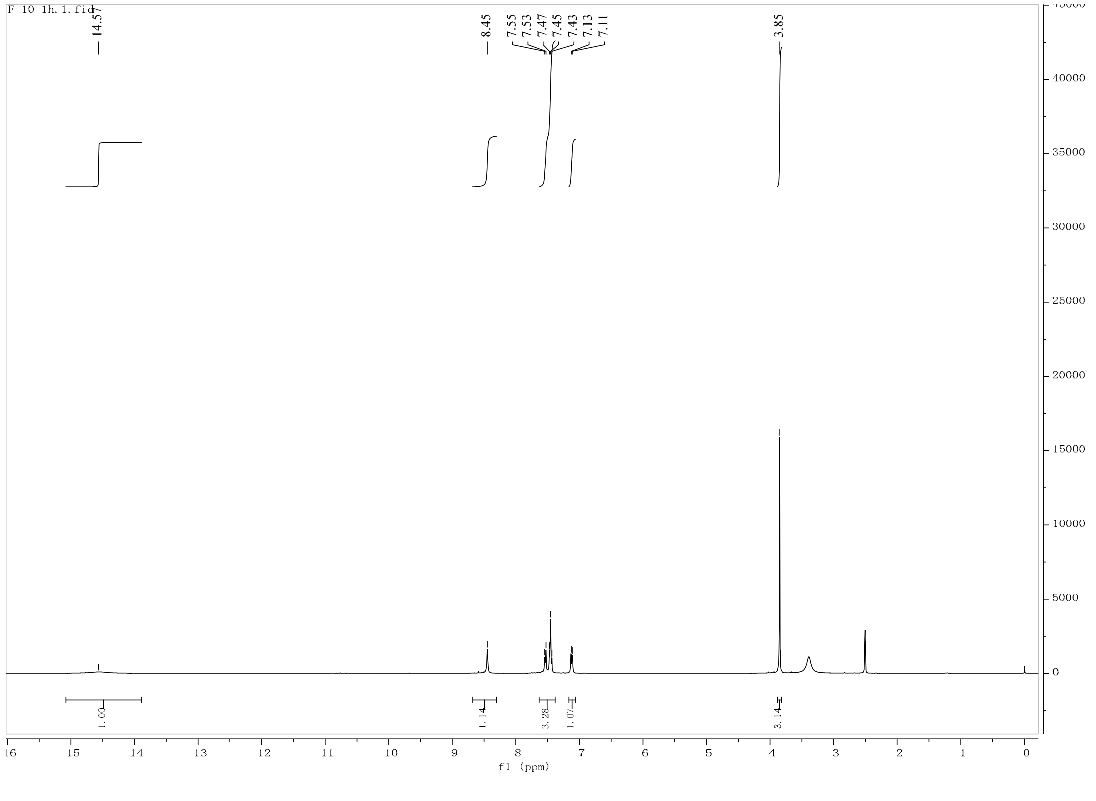

The ^{1}H NMR spectrogram of compound **5j**

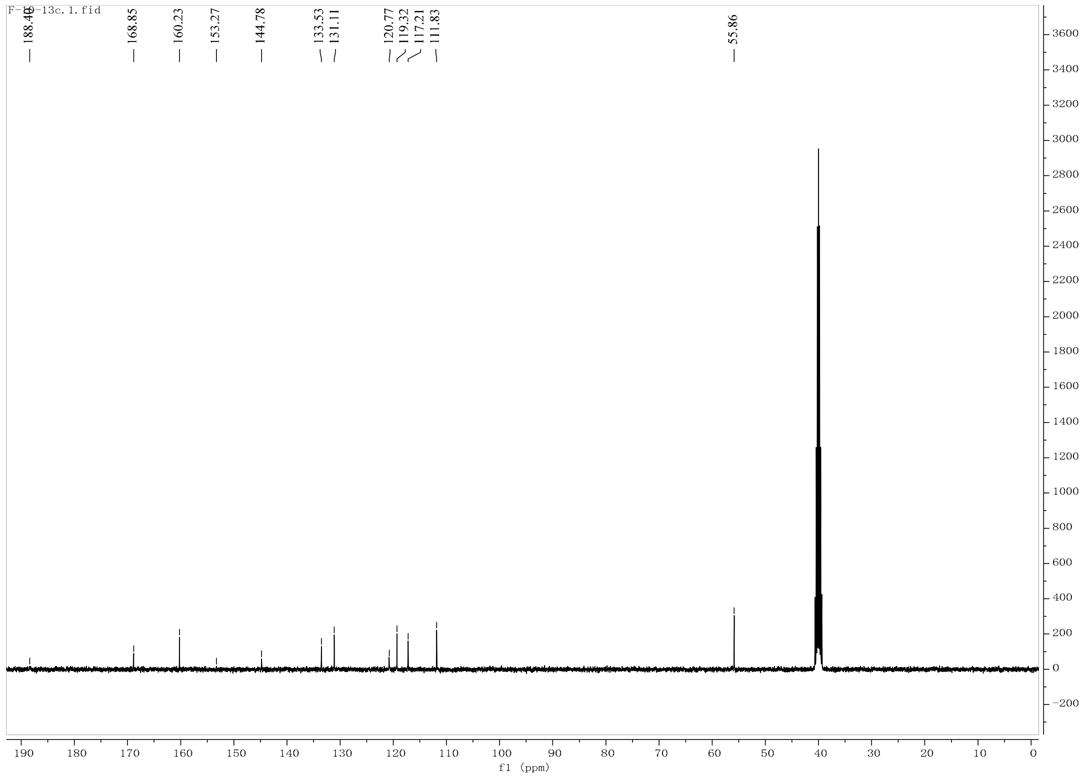

The ^{13}C NMR spectrogram of compound **5j**

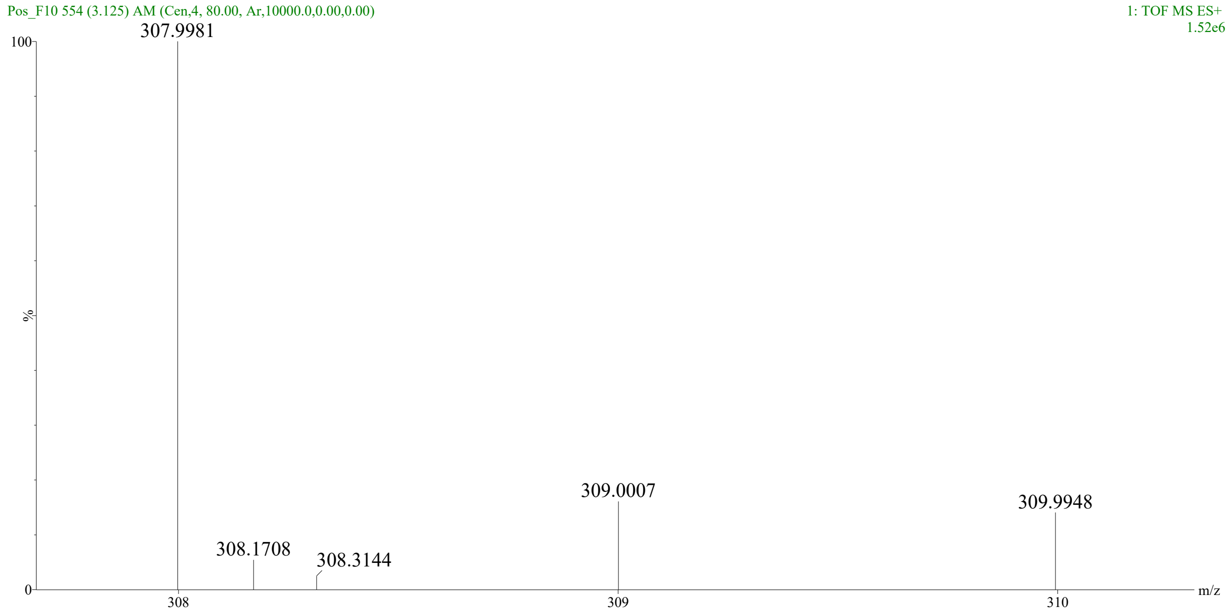

The HRMS spectrogram of compound **5j**

Compound **5k**, 5-(2-(3-(trifluoromethoxy)phenyl)thiazol-4-yl)-1,3,4-thiadiazole-2(3*H*)-thione:

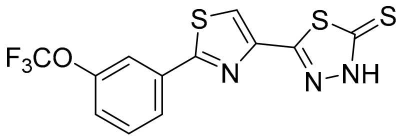

white solid, yield 82.6 %, m. p. 228.6-228.9°C; ^{1}H NMR (400 MHz, DMSO-d_6) δ: 14.80 (s, 1H), 8.51 (s, 1H), 8.00 (d, J = 8.3 Hz, 1H), 7.90 (s, 1H), 7.68 (t, J = 8.0 Hz, 1H), 7.59 – 7.52 (m, 1H). ^{13}C NMR (101 MHz, DMSO-d_6) δ: 187.91, 166.63, 154.65, 148.88 (d, J = 2.0 Hz), 144.47, 133.85, 131.66, 125.64, 123.21, 121.13, 120.02 (q, J = 256.9 Hz), 118.43. HRMS (ESI): calcd for $C_{12}H_6F_3N_3OS_3$[M+H]$^+$: 361.9698, found,

361.9698.

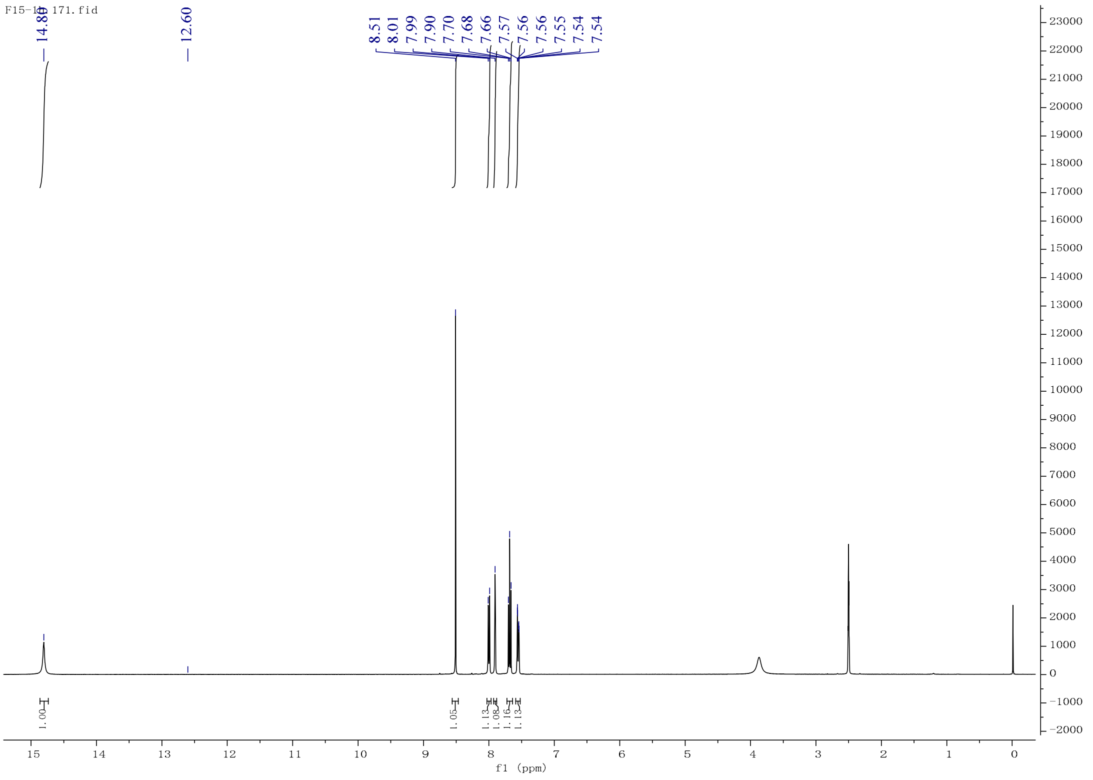

The ^{1}H NMR spectrogram of compound **5k**

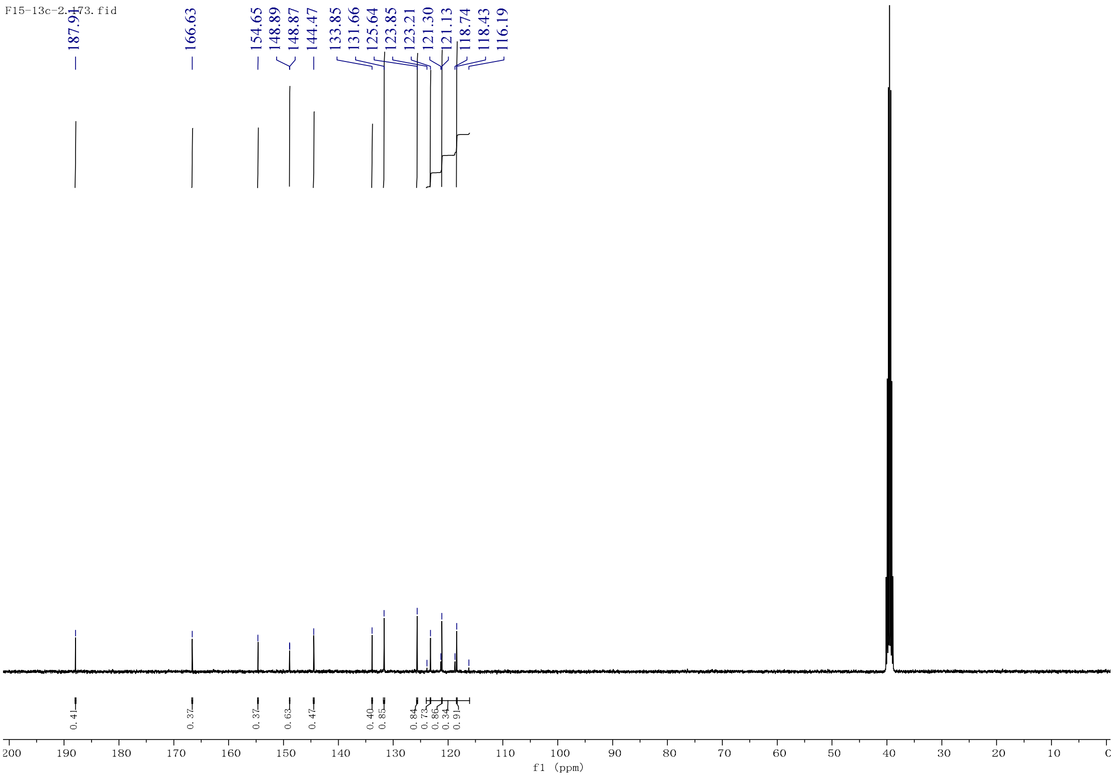

The ^{13}C NMR spectrogram of compound **5k**

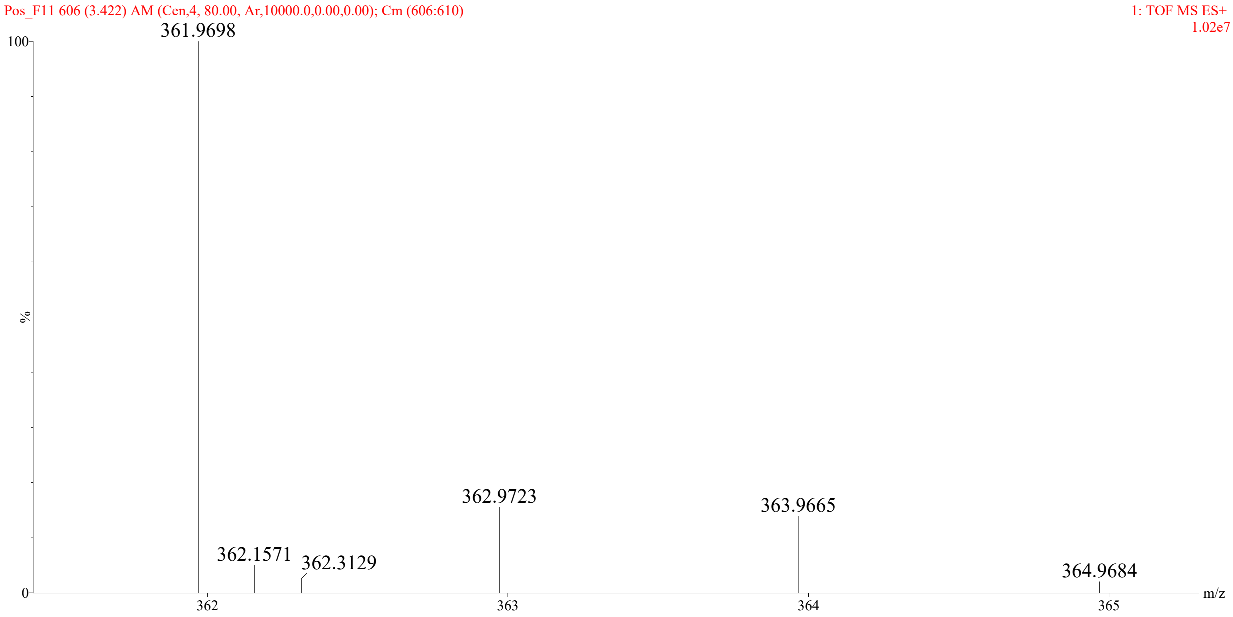

The HRMS spectrogram of compound **5k**

Compound **5l**, 5-(2-(4-fluorophenyl)thiazol-4-yl)-1,3,4-thiadiazole-2(3*H*)-thione: 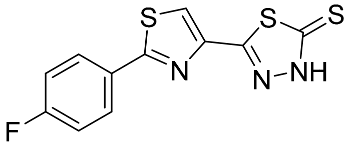

white solid, yield 81.8 %, m. p. >250°C; ^{1}H NMR (400 MHz, DMSO-d_6) δ: 14.75 (s, 1H), 8.39 (s, 1H), 8.09 – 7.82 (m, 2H), 7.34 (t, *J* = 8.8 Hz, 2H). ^{13}C NMR (101 MHz, DMSO-d_6) δ: 187.87, 167.41, 163.62 (d, *J* = 249.3 Hz), 154.83, 144.30, 128.79 (d, *J* = 8.8 Hz), 128.48 (2C) (d, *J* = 3.0 Hz), 120.12, 116.43 (2C) (d, *J* = 22.3 Hz). HRMS (ESI): calcd for $C_{11}H_6FN_3S_3$ [M+H]$^+$: 295.9781, found, 295.9781.

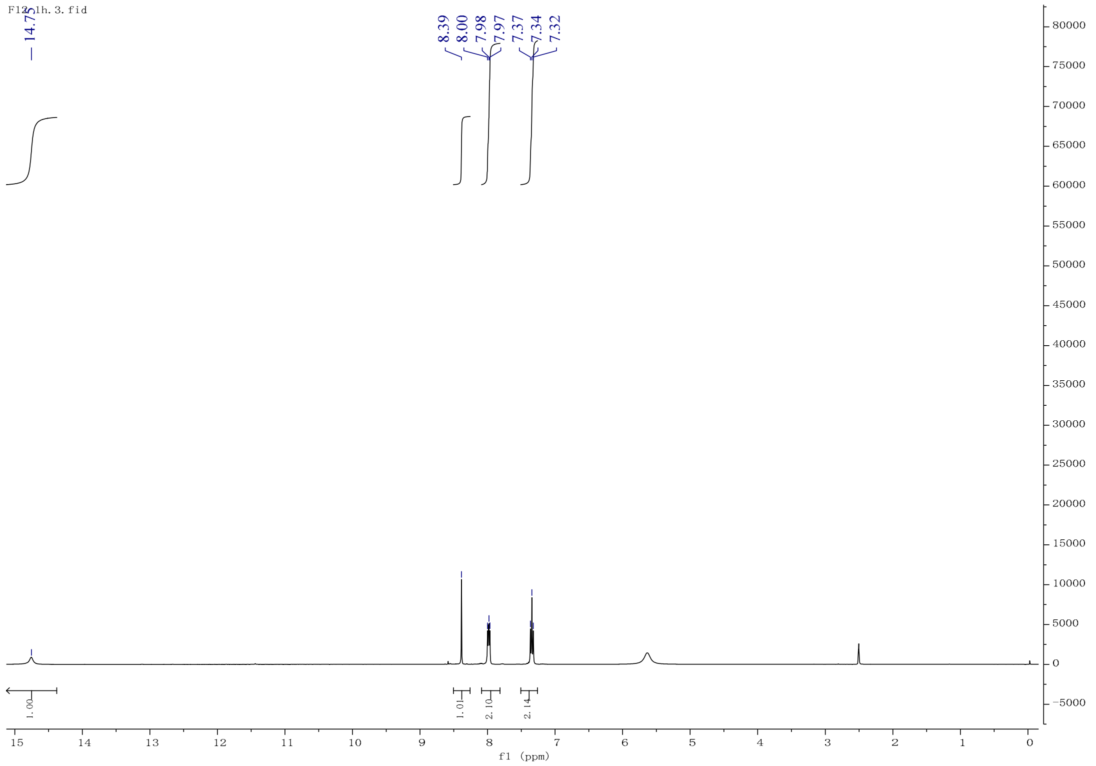

The ^{1}H NMR spectrogram of compound **5l**

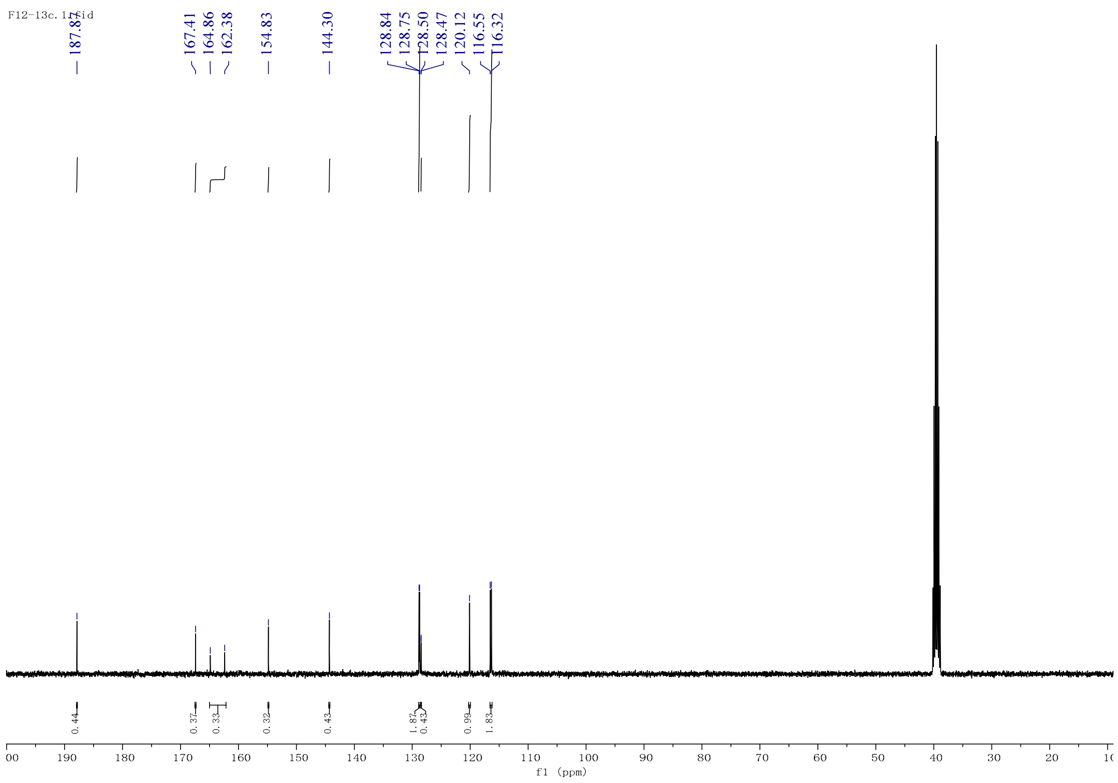

The ^{13}C NMR spectrogram of compound **5l**

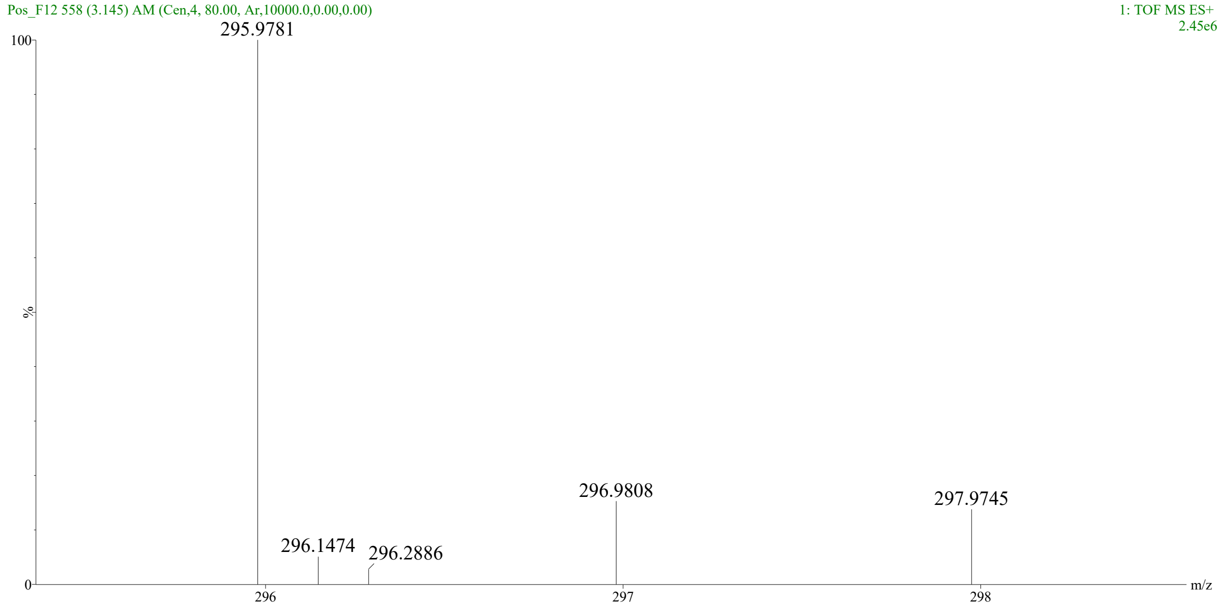

The HRMS spectrogram of compound **5l**

Compound **5m**, 5-(2-(p-tolyl)thiazol-4-yl)-1,3,4-thiadiazole-2(3*H*)-thione:

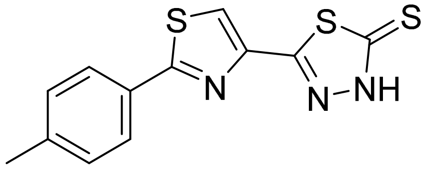

yellow solid, yield 85.2 %, m. p.247.7-248.6°C; ^{1}H NMR (400 MHz, DMSO-d_6) δ: 14.76 (s, 1H), 8.33 (s, 1H), 7.80 (d, J = 8.1 Hz, 2H), 7.30 (d, J = 8.0 Hz, 2H), 2.34 (s, 3H). ^{13}C NMR (101 MHz, DMSO-d_6) δ: 188.30, 169.18, 155.46, 144.65, 141.54, 130.34 (2C), 129.65, 126.73 (2C), 119.96, 21.47. HRMS (ESI): calcd for $C_{12}H_9N_3S_3$

[M+H]$^+$: 292.0032, found, 292.0033.

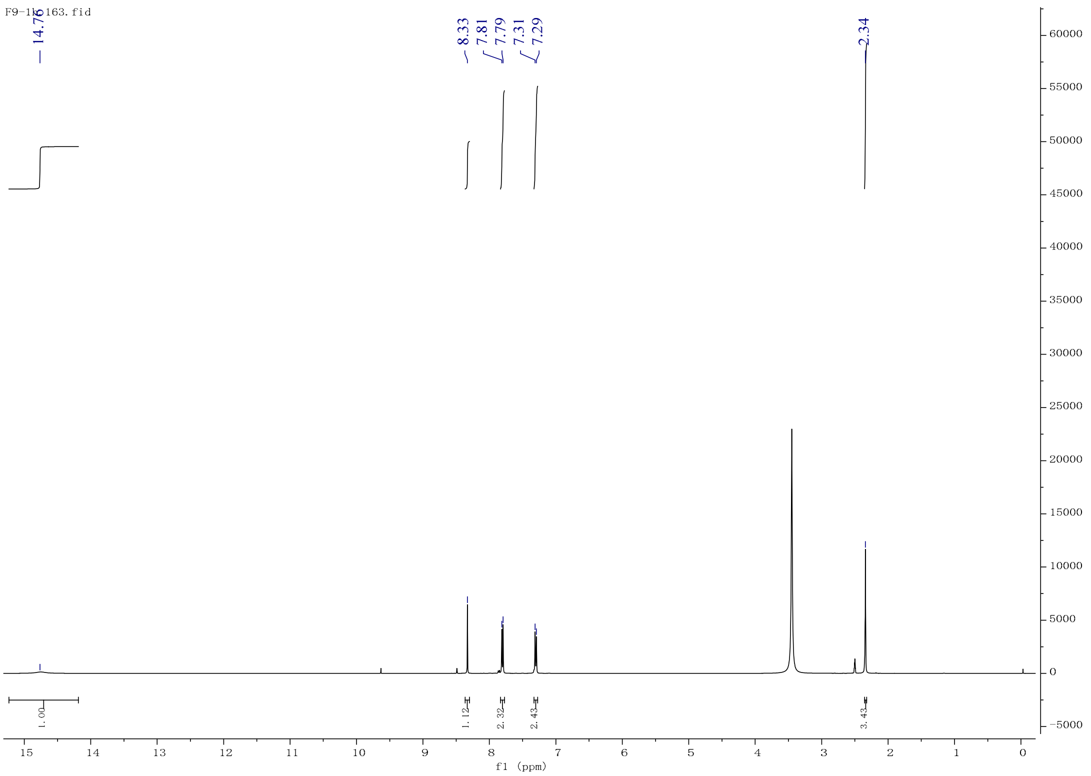

The ^{1}H NMR spectrogram of compound **5m**

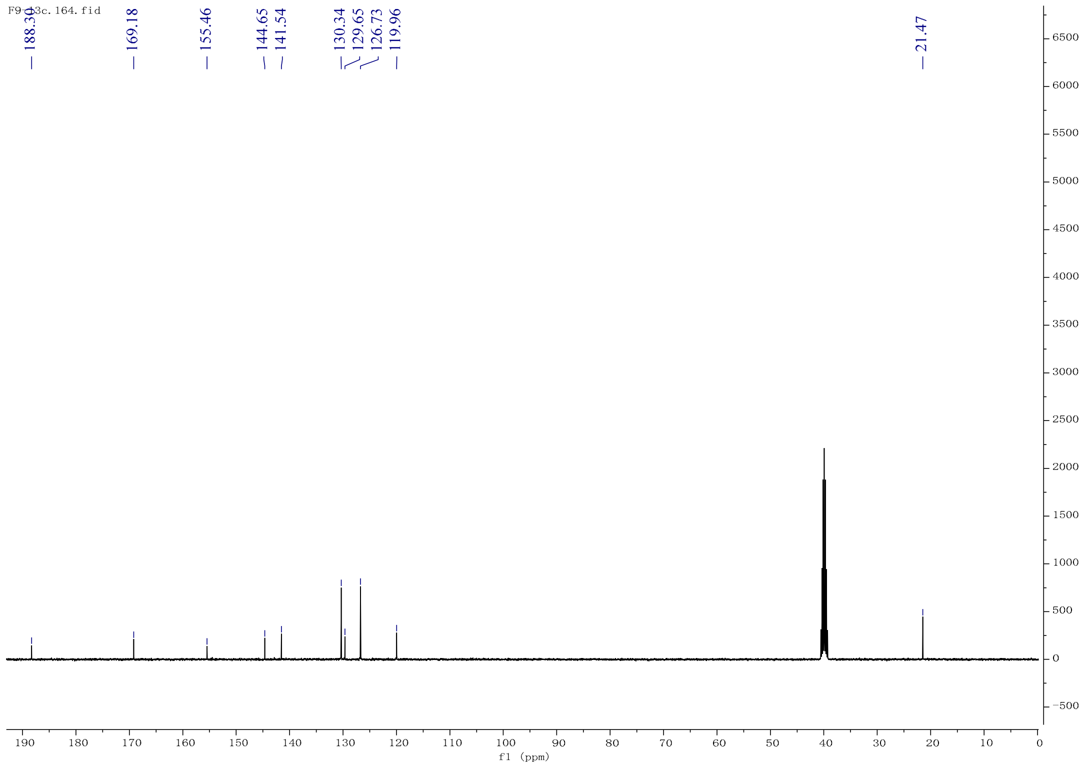

The ^{13}C NMR spectrogram of compound **5m**

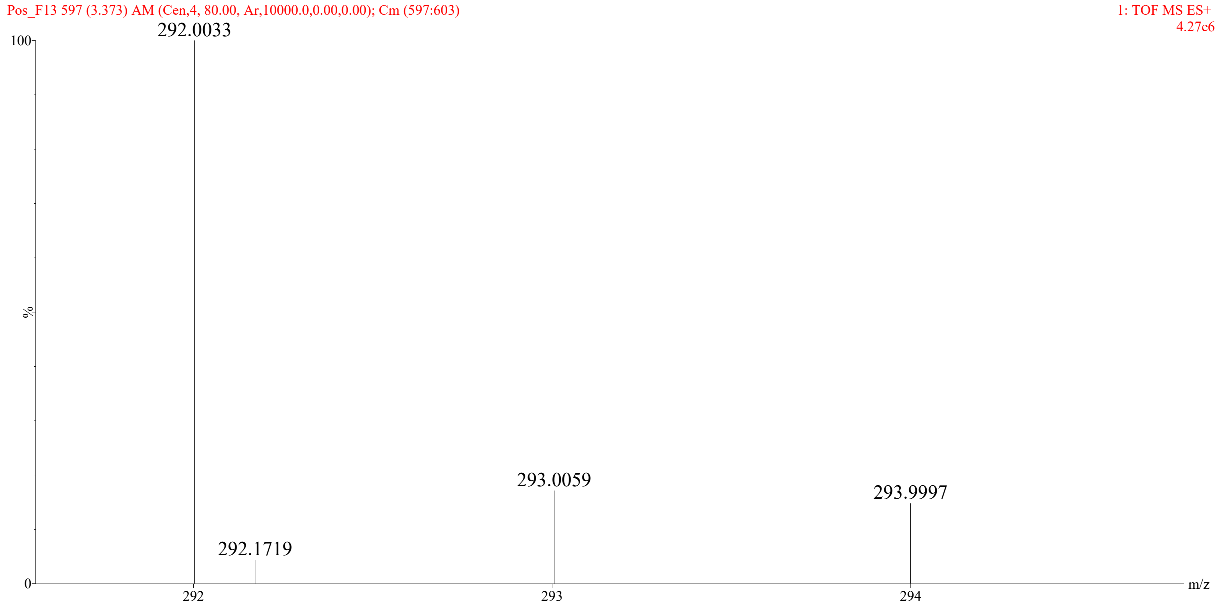

The HRMS spectrogram of compound **5m**

Compound **5n**, 5-(2-(4-methoxyphenyl)thiazol-4-yl)-1,3,4-thiadiazole-2(3*H*)-thione: 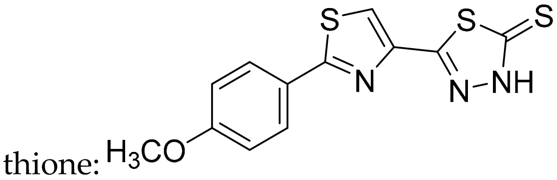

yellow solid, yield 82.1 %, m. p. 219.8-221.5°C; ^{1}H NMR (400 MHz, DMSO-d_6) δ: 14.76 (s, 1H), 8.41 (d, J = 57.9 Hz, 1H), 7.95 (d, J = 8.8 Hz, 1H), 7.90 (d, J = 8.8 Hz, 1H), 7.11 – 7.06 (m, 2H), 3.83 (s, 3H). ^{13}C NMR (101 MHz, DMSO-d_6) δ: 188.19, 168.97, 163.10, 161.89, 154.39, 145.69, 144.58, 125.08 (2C), 119.55 (2C), 55.93. HRMS (ESI): calcd for $C_{12}H_9N_3OS_3$ [M+H]$^+$: 307.9981, found, 307.9981.

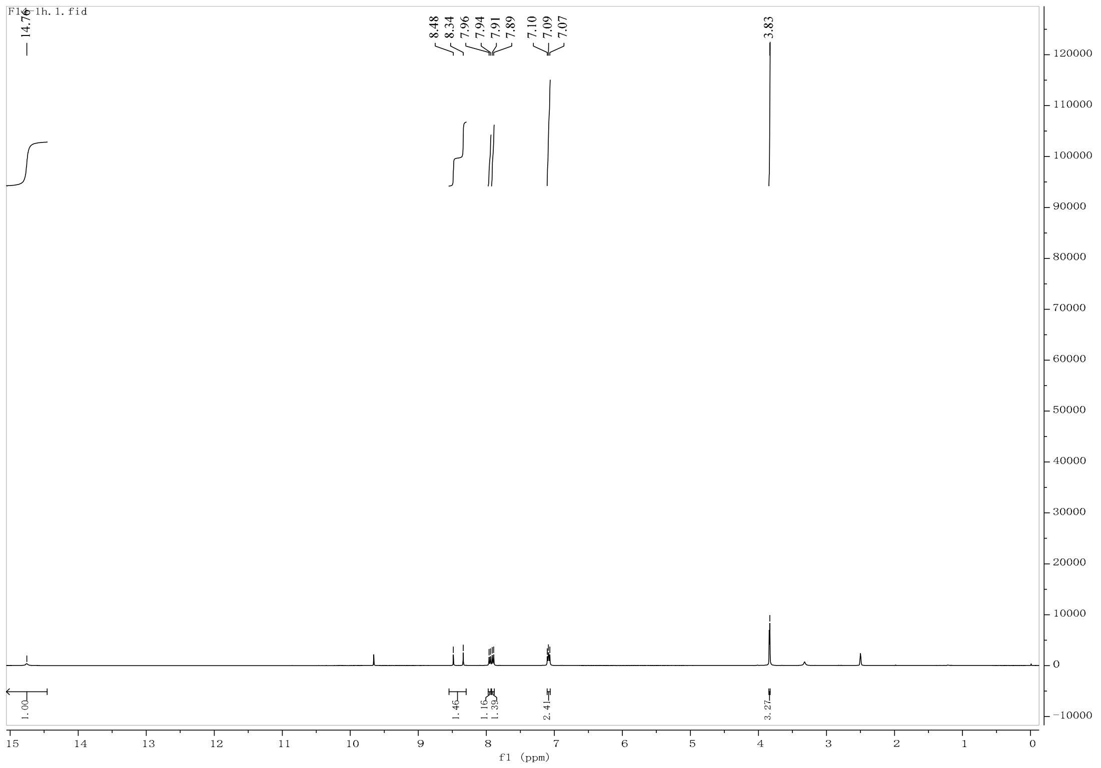

The ^{1}H NMR spectrogram of compound **5n**

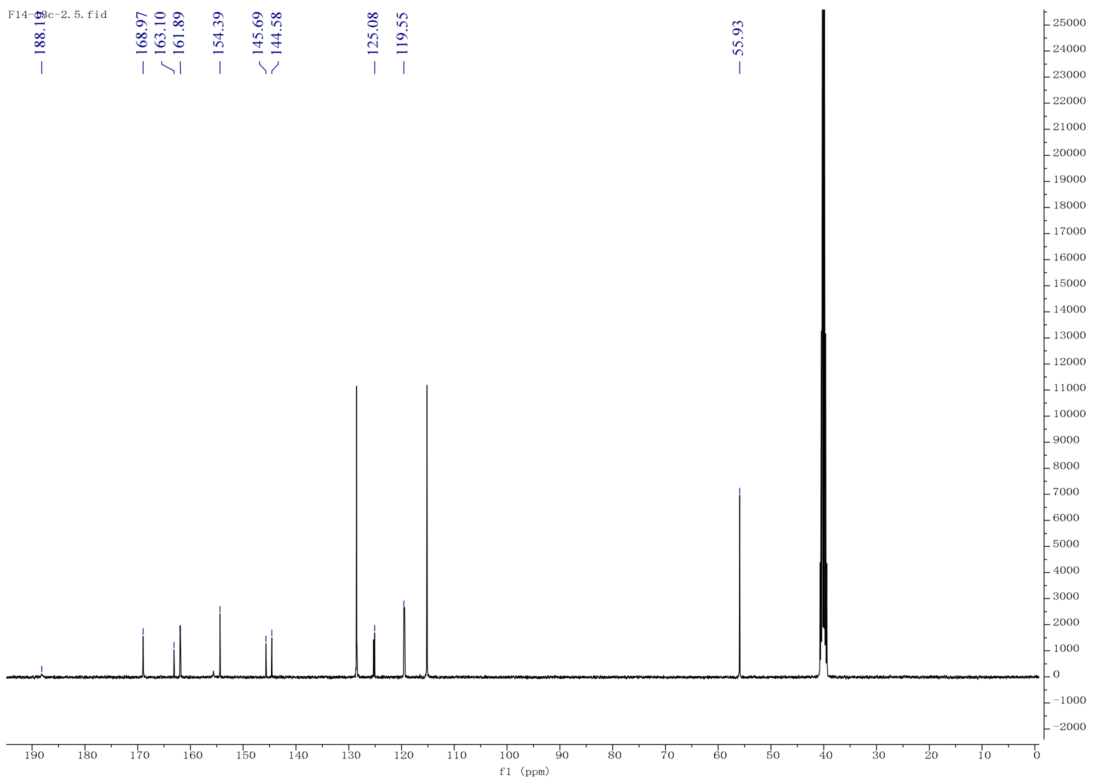

The ^{13}C NMR spectrogram of compound **5n**

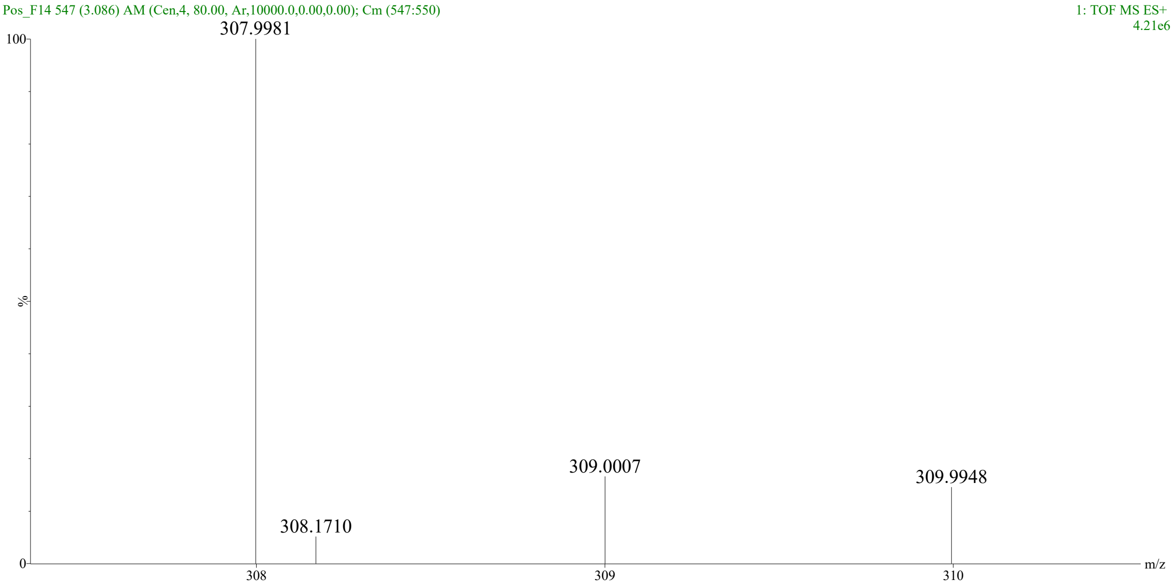

The HRMS spectrogram of compound **5n**

Compound **5o**, 5-(2-(4-(trifluoromethoxy)phenyl)thiazol-4-yl)-1,3,4-thiadiazole-2(3*H*)-thione:

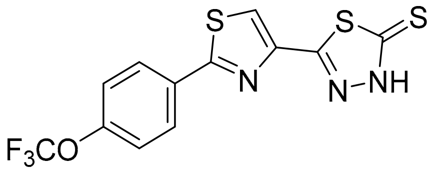

white solid, yield 83.5 %, m. p. 246.8-247.6°C; ^{1}H NMR (400 MHz, DMSO-d_6) δ: 14.75 (s, 1H), 8.42 (s, 1H), 8.03 (d, J = 8.7 Hz, 2H), 7.47 (d, J = 8.3 Hz, 2H). ^{13}C NMR (101 MHz, DMSO-d_6) δ: 187.88, 166.88, 154.69, 149.90, 144.48, 130.84, 128.43 (2C), 121.62 (2C), 120.62, 119.94 (q, J = 257.4 Hz). HRMS (ESI): calcd for $C_{12}H_6F_3N_3OS_3$ [M+H]$^+$: 361.9698, found, 361.9698.

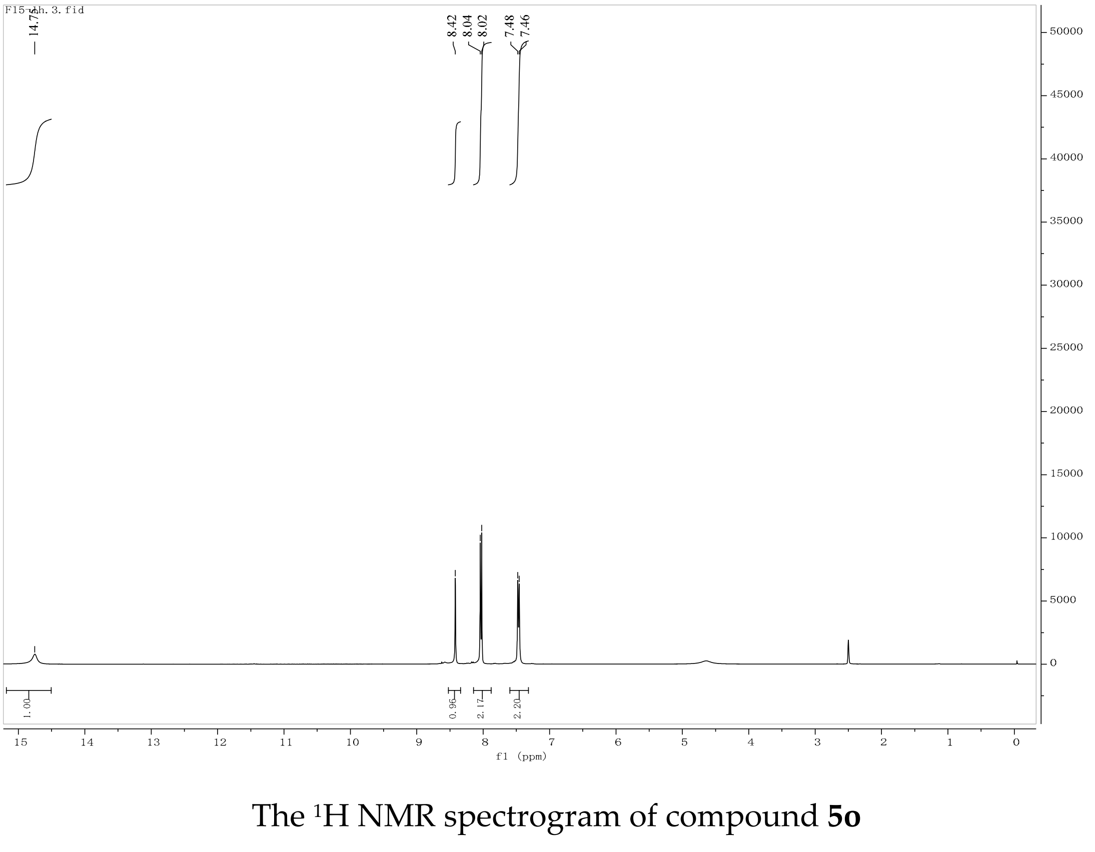

The ^{1}H NMR spectrogram of compound **5o**

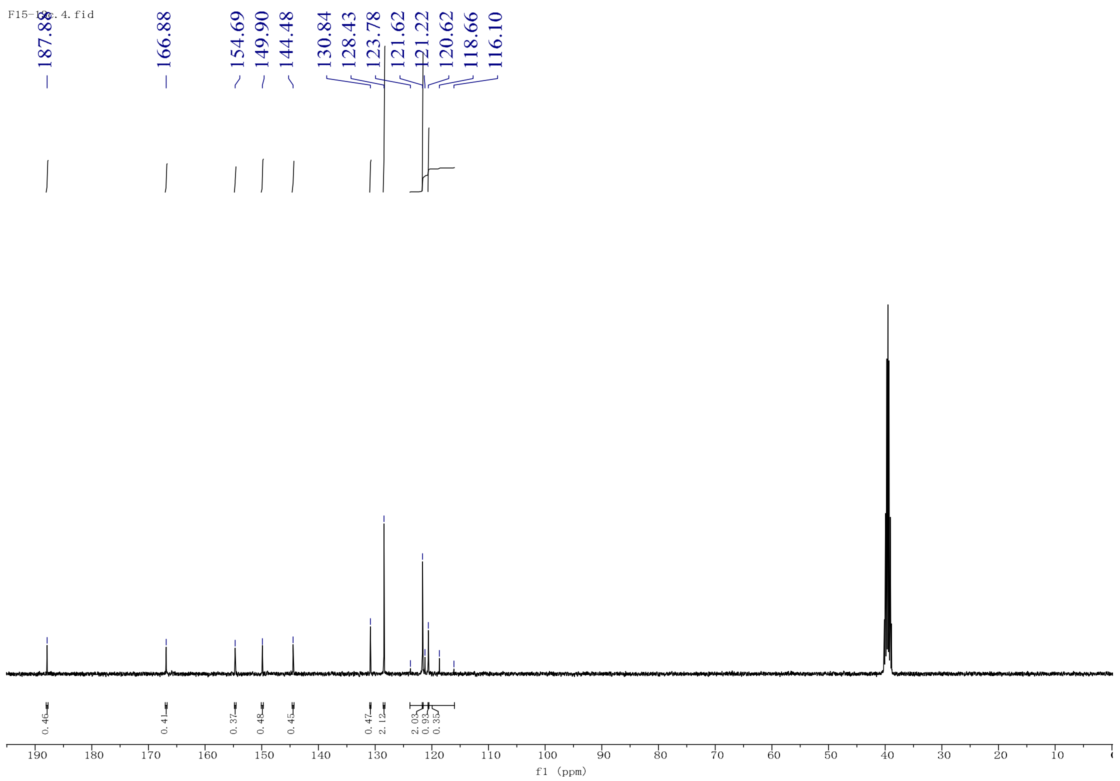

The ^{13}C NMR spectrogram of compound **5o**

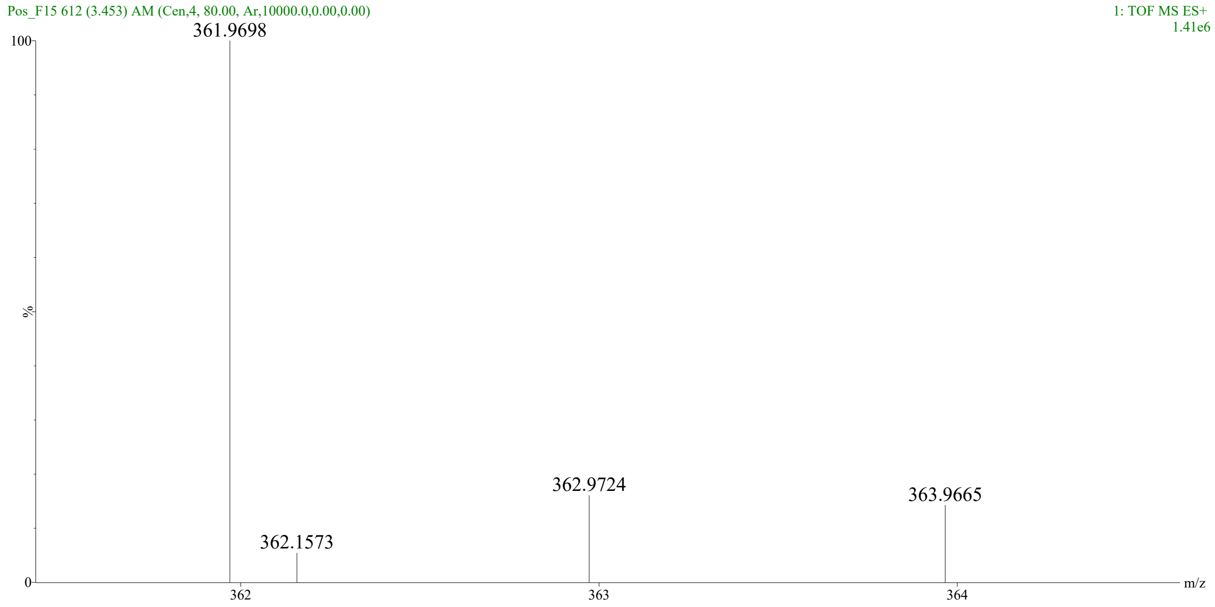

The HRMS spectrogram of compound **5o**

Compound **5p**, 5-(2-(4-aminophenyl)thiazol-4-yl)-1,3,4-thiadiazole-2(3*H*)-thione:

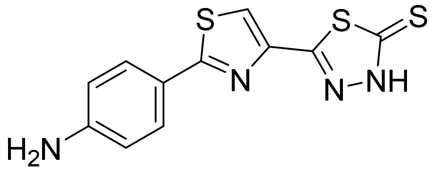

gray solid, yield 86.4 %, m. p. >250°C; ^{1}H NMR (400 MHz, DMSO-d_6) δ: 14.85 (s, 1H), 8.33 (s, 1H), 7.59 (dd, J = 8.0, 1.3 Hz, 1H), 7.19 (t, J = 7.7 Hz, 1H), 6.87 (d, J = 8.3 Hz, 2H), 6.63 (t, J = 7.5 Hz, 2H). ^{13}C NMR (101 MHz, DMSO-d_6) δ: 188.08, 170.58, 155.20, 146.97, 143.53, 132.09, 129.52, 118.18, 117.23, 116.39, 113.28. HRMS (ESI): calcd for $C_{11}H_8N_4S_3$ [M+H]$^+$:292.9984, found, 292.9985.

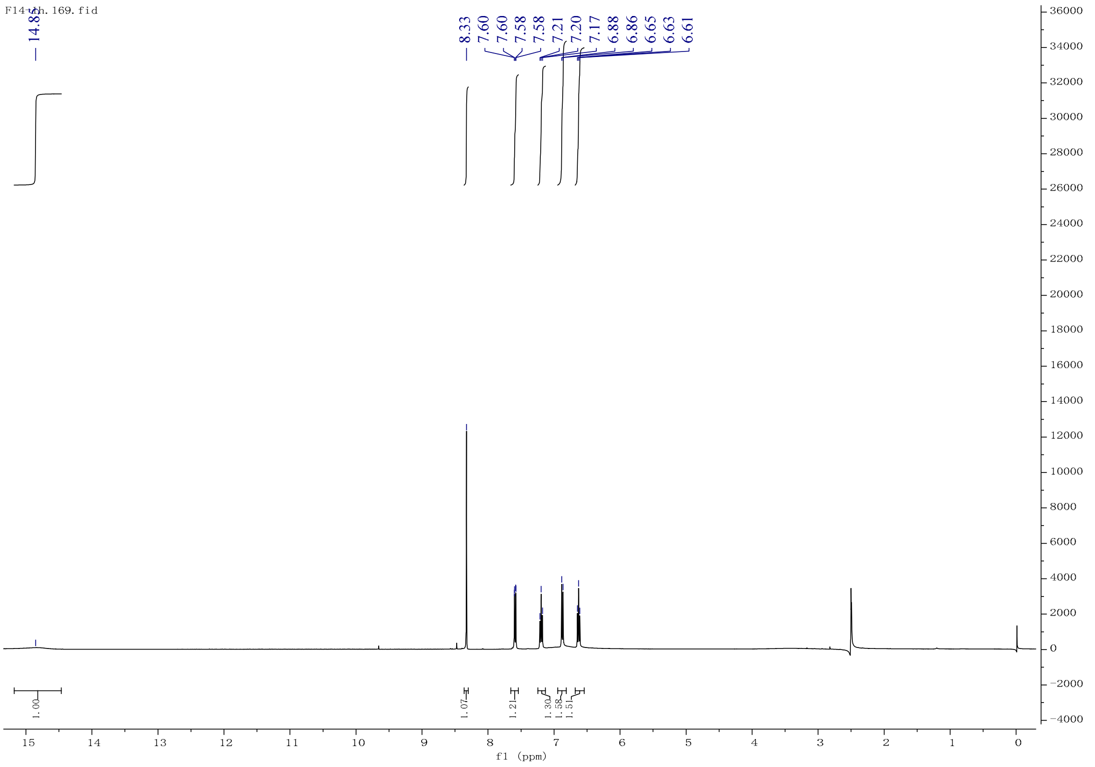

The ^{1}H NMR spectrogram of compound **5p**

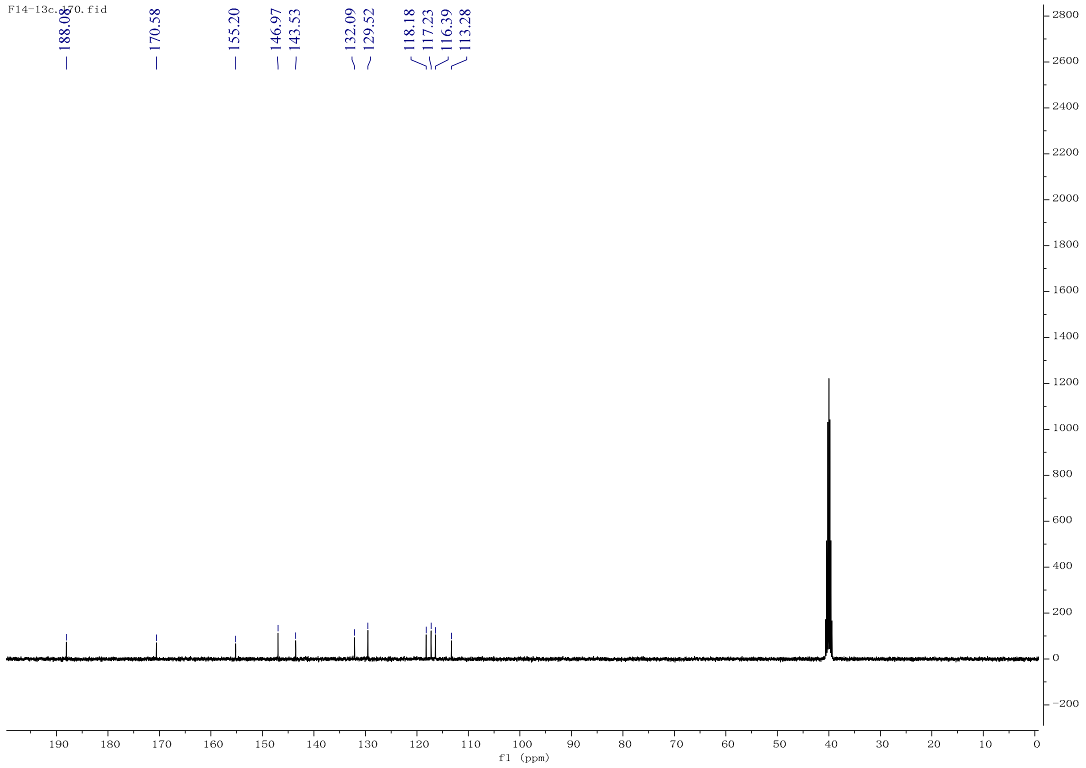

The ^{13}C NMR spectrogram of compound **5p**

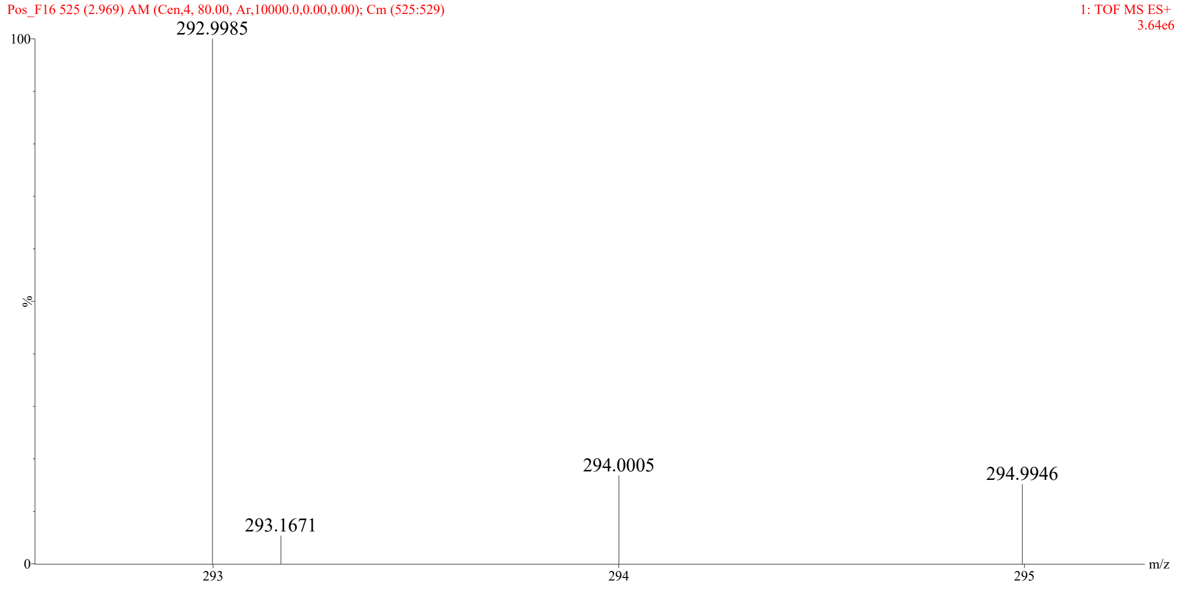

The HRMS spectrogram of compound **5p**
